# Inhibition of pyrimidine *de novo* synthesis fosters Treg cells and reduces diabetes development in models of Type 1 Diabetes

**DOI:** 10.1016/j.molmet.2025.102218

**Published:** 2025-07-22

**Authors:** Hannah Hipp, Camilla Tondello, Hanna Gmehling, Lena K. Scholz, Antigoni Stavridou, Maike Becker, Anne-Marie Bührer, Edith Hintermann, Sandra M. Dirschl, Till M. Johannsmann, Martin G. Scherm, Hella Kohlhof, Isabelle Serr, Urs Christen, Carolin Daniel

**Affiliations:** 1 Research Unit Type 1 Diabetes Immunology, Helmholtz Diabetes Center at Helmholtz Zentrum München, Munich, Germany; 2 Deutsches Zentrum für Diabetesforschung (DZD), Munich, Germany; 3 Pharmazentrum Frankfurt, Goethe University Frankfurt, Frankfurt am Main, Germany; 4 Center for Regenerative Therapies Dresden (CRTD), Technische Universität Dresden, Dresden, Germany; 5 Faculty of Life Sciences and Medicine, Department of Immunobiology, King's College London, London, UK; 6 Immunic AG, Gräfelfing, Germany; 7 Division of Clinical Pharmacology, Department of Medicine IV, Ludwig-Maximilians-Universität München, Munich, Germany

**Keywords:** Autoimmunity, Type 1 diabetes, Regulatory T cells, Immunomodulation, DHODH inhibition, Vidofludimus calcium

## Abstract

**Objective:**

In autoimmune Type 1 Diabetes (T1D), aberrant immune activation promotes regulatory T cell (Treg) impairments thereby boosting progression of islet autoimmunity. Consequently, there is a progressive destruction of the insulin-producing beta cells in the pancreas. Controlling overshooting immune activation represents a relevant approach to allow for efficient Treg-targeting by broadening the window of opportunity to induce Tregs.

**Methods:**

We investigated the effect of restricting pyrimidine *de novo* synthesis during islet autoimmunity and T1D by Dihydroorotate dehydrogenase (DHODH) inhibition using the next-generation DHODH inhibitor Vidofludimus calcium. We assessed Treg-inducing features of DHODH inhibition in T cells from ongoing murine islet autoimmunity and human T1D *in vitro*. To dissect the functional relevance of these observations, we tested the impact of DHODH inhibition on interfering with autoimmune activation and disease progression in pre-clinical models of T1D *in vivo*.

**Results:**

We show that DHODH inhibition results in enhanced Treg induction *in vitro* especially during increased immune activation and reduced T cell proliferation. In addition, Vidofludimus calcium reduced T1D incidence in two mouse models. On the cellular level, treated mice showed reduced T cell activation accompanied by increased Treg frequencies.

**Conclusions:**

We demonstrate that restricting pyrimidine *de novo* synthesis by next-generation DHODH inhibition is a strategy to interfere with autoimmune activation while fostering Tregs.

## Highlights

• Aberrant immune activation promotes regulatory T cell (Treg) impairments.
• DHODH inhibition enhances Treg induction *in vitro* during immune activation.
• DHODH inhibition delays disease development in type 1 diabetes (T1D) models.
• Treated mice show reduced T cell activation and increased Treg frequencies.
• DHODH inhibition interferes with autoimmune activation while fostering Tregs.

## Graphical abstract

**Figure:**
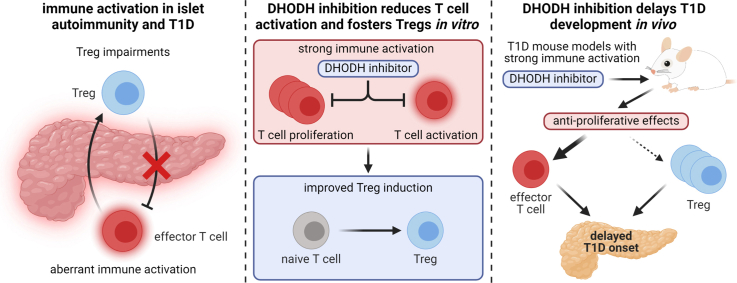

## 1 Introduction

Type 1 Diabetes (T1D) is an organ specific autoimmune disease and shows rising incidences around the world, especially in young children [1]. The disease is characterized by the autoimmune-mediated destruction of the insulin producing beta cells in the pancreas, resulting from a breakdown of immune tolerance and aberrations in immune activation [2]. Prior to the manifestation of clinical symptoms, T1D patients develop multiple autoantibodies which are specific for islet autoantigens such as insulin [3]. This pre-symptomatic phase of islet-autoimmunity is termed stage 1 T1D. In stage 2, which is still pre-symptomatic, the progressive destruction of beta cells leads to the development of dysglycemia. Stage 3 is defined as the onset of clinical symptoms of T1D [4]. Despite advances in insulin therapies, children who develop T1D before the age of ten years, still face an expected loss of 16 life years [5]. Therefore, there is a high medical need for immune therapies that can decelerate or even halt autoimmune activation and progression. In a recent study, the treatment of at-risk individuals with stage 2 diabetes with the anti-CD3 antibody teplizumab delayed the median time to onset of stage 3 T1D by 2.7 years [6]. Accordingly, teplizumab was recently approved by the Food and Drug Administration (FDA) for pre-symptomatic stage 2 T1D [7]. This successful development highlights the relevance of immunomodulatory strategies to delay T1D development while also emphasizing the need for tailored immune modulators with minimized characteristics of general immunosuppressive agents.

Regulatory T (Treg) cells are critical cellular mediators of immune tolerance [8]. Tregs are characterized by the expression of the high-affinity interleukin-2 (IL-2) receptor α-chain (IL-2Rα, CD25) and the lineage specifying transcription factor forkhead box P3 (Foxp3) [[8], [9], [10]]. Foxp3 expression is crucial for Treg development and functional identity [8,10]. We identified multiple layers of aberrations in the induction and stability of Tregs as key drivers of islet autoimmune activation and the progression to clinical T1D [11,12]. Accordingly, in states of increased T cell activation, such as during islet autoimmunity, human and murine Treg induction *in vitro* is severely impaired [11,12]. Therefore, there is an urgent need of immunomodulatory strategies that are suited to foster Treg induction during states of aberrant immune activation. Interfering with aberrant immune activation could open a window of opportunity for efficient Treg induction during ongoing islet autoimmunity. Conceptually, the reduction of immune activation could be achieved by inhibition of the *de novo* pyrimidine biosynthesis, which is essential for proliferating and activated T cells due to their increased pyrimidine demand [[13], [14], [15]].

Dihydroorotate dehydrogenase (DHODH) catalyses the rate-limiting step of *de novo* pyrimidine biosynthesis and therefore provides a promising target for inhibitors [13,16]. Vidofludimus calcium (VidoCa, IMU-838; Immunic AG, Germany) is a selective and potent second generation oral DHODH inhibitor. The structure of IMU-838 is unrelated to classical DHODH inhibitors such as leflunomide (Lef) or teriflunomide (Tef) [15]. Accordingly, IMU-838 did not show any adverse events attributed to general anti-proliferative effects of Lef and Tef resulting from off-target effects [15,[17], [18], [19]]. Previous studies demonstrated that DHODH inhibition using IMU-838 leads to reduced T cell proliferation, reduced pro-inflammatory cytokine release and increased apoptosis of activated lymphocytes [15]. IMU-935 (Immunic AG, Germany) inhibits DHODH to a lesser extent than IMU-838 while it also functions as a RORγt inverse agonist [20,21]. RORγt is the T_H_17 cell-defining transcription factor and is, together with Foxp3, also upregulated in Treg-skewing conditions with TGF-β [22]. T_H_17-like Tregs express both master transcription factors, Foxp3 and RORγt [23,24]. Synergistic inhibition of RORγt and DHODH suppressed T_H_17 differentiation and the release of pro-inflammatory cytokines including IL-17A, IL-17F and IFN-γ, dampening inflammatory responses and autoimmune activation [21,25]. In murine models for psoriasis and colitis, treatment with IMU-935 showed relevant therapeutic activity [21,25]. Likewise, DHODH inhibition by IMU-838 or Vidofludimus free acid improved disease outcomes in preclinical models of autoimmune diseases such as colitis [26] and experimental autoimmune encephalomyelitis (EAE) [27]. Recent evaluation of the EMPhaSIS trial, a phase 2 trial involving relapsing-remitting multiple sclerosis (RRMS) patients, confirmed preclinical data with a reduction in new magnetic resonance imaging lesions and reported a favorable safety and tolerability profile [17]. Administration of IMU-935 in a first-in-human phase 1 study was safe and did not show any dose-limiting toxicity [20].

Despite these insights, the impact of RORγt inverse agonism and especially second generation DHODH inhibition on Treg induction and function during states of ongoing islet-autoimmune activation remains currently unexplored. To fill this knowledge gap, in the present study we assessed Treg-inducing features of IMU-838 and IMU-935 in T cells from ongoing murine islet autoimmunity and human T1D *in vitro*. To dissect the functional relevance of these observations, we tested the impact of IMU-838 on interfering with autoimmune activation and disease progression in pre-clinical models of T1D *in vivo*. Our results show that DHODH inhibition can control aberrant immune activation while fostering Tregs even in settings of strong immune activation. Importantly, using two unrelated murine models of T1D in two independent laboratories, DHODH inhibition using IMU-838 significantly reduced T1D incidences.

## 2 Results

### 2.1 IMU-935 enhances human Treg induction *in vitro* under conditions of strong immune activation while reducing T cell proliferation

**Figure 1:**
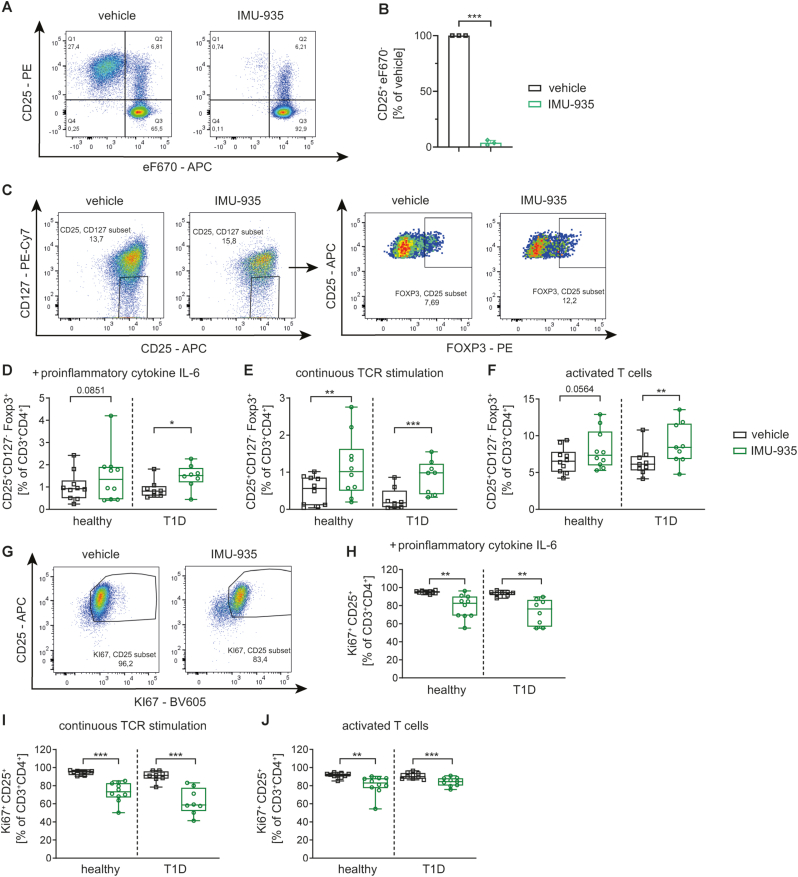
**IMU-935 improves human Treg induction *in vitro* during conditions of increased immune activation.** (**A, B**) Human T cell proliferation assay to assess the CD4^+^ T cell response to Influenza (flu) vaccine using PBMCs from healthy blood donors. (**A**) Representative FACS staining of the proliferation of CD4^+^ T cells in presence of 5 nM IMU-935 or the respective vehicle control. (**B**) Summary plots of proliferating CD25^+^CD4^+^ T cells normalized to the vehicle control, n = 3. (**C–F**) Human Treg induction *in vitro* during conditions of increased immune activation using naïve or activated T cells from PBMCs of healthy subjects and T1D donors in presence of 3.5 nM IMU-935 or the respective vehicle control. (**C**) Representative FACS staining of Treg induction *in vitro* using continuous TCR stimulation in presence or absence of IMU-935. Summary plots of (**D**) limited TCR stimulation using naïve T cells in presence of the pro-inflammatory cytokine IL-6 or (**E**) continuous TCR stimulation. (**F**) Frequency of induced CD25^+^CD127^−^FOXP3^+^ Tregs upon Treg induction using activated T cells, healthy n = 10, T1D n = 8. (**G-J**) Proliferating Ki67^+^CD25^+^ T cells in presence of 3.5 nM IMU-935 or the respective vehicle control after Treg induction during immune activating conditions. (**G**) Representative FACS staining. Summary plots of Ki67^+^CD25^+^ T cells frequency after Treg induction using (**H**) subimmunogenic TCR stimulation and naïve T cells in presence of the pro-inflammatory cytokine IL-6, (**I**) continuous TCR stimulation or (**J**) Treg induction using activated T cells, healthy n = 10, T1D n = 8. Each data point represents one donor, experiments were performed in 2–3 technical replicates. Student's t test ∗ = p < 0.05; ∗∗ = p < 0.01; ∗∗∗ = p < 0.001; ∗∗∗∗ = p < 0.0001.

We first analyzed the impact of IMU-935 on the response of human CD4^+^ T cells to various antigens. As antigenic stimulation we chose the model antigen influenza vaccine since it can activate high percentages of antigen-specific CD4^+^ T cells due to high prevalence of influenza infection or vaccination. IMU-935 prominently reduced the response of antigen-specific CD4^+^ T cells to influenza antigens, as evidenced by a reduced proliferation (Figure 1A,B, Fig. S1A). Similarly, we also assessed the response to the super-antigen SEB (Staphylococcal enterotoxin B) which can stimulate a large fraction of T cell receptors (TCR) by binding with high affinity to TCRVβ17. Stimulation of naïve and memory human CD4^+^ T cells with SEB in presence of IMU-935 resulted in reduced responses of both subsets of TCRVβ17-specific T cells to SEB (Fig S1, B-E).

Decreased T cell proliferation and activation is well known to be beneficial for *de novo* Treg induction *in vitro*. Thus, next we investigated the immunomodulatory effect of IMU-935 on human T cells. Subimmunogenic TCR stimulation provides optimal conditions for inducing Tregs *in vitro*. Under these conditions, IMU-935 increased the frequencies of induced CD25^+^CD127^−^Tregs using naïve CD3^+^CD4^+^CD45RA^+^CD45RO^−^CD25^−^CD127^+^ T cells from both healthy donors and those with T1D (Fig. S2, A‒C). Furthermore, the T cell proliferation marker Ki67 was reduced by IMU-935 (Fig. S2D).

Next, we investigated whether IMU-935 fosters the induction of human Tregs *in vitro* under conditions of strong immune activation, mirroring those seen in islet autoimmunity and T1D. To mimic conditions of aberrant immune activation *in vitro*, Tregs were induced from naïve CD4^+^ T cells using subimmunogenic TCR stimulation in presence of the pro-inflammatory cytokine IL-6 or using continuous TCR stimulation. In addition, Tregs were induced from human T cells with a previously activated memory phenotype (CD3^+^CD4^+^CD45RA^−^CD45RO^+^CD25^−^CD127^+^ (Fig. S2A)). Activated T cells show a transient upregulation of FOXP3 [28]. Therefore, we excluded contamination of any FOXP3^+^ cells in sorted activated T cells by post-sort FOXP3 staining (Fig. S2E). IMU-935 significantly improved the induction of CD25^+^CD127^−^FOXP3^+^ Tregs using T cells isolated from peripheral blood of T1D patients in all three experimental conditions (Figure 1C–F). Using T cells from healthy donors, IMU-935 significantly enhanced Treg induction using continuous TCR stimulation. In the other conditions, there was a trend towards improved Treg induction (Figure 1C–F). Moreover, under conditions of continuous stimulation, we assessed the functional state of IMU-935-induced CD25^+^CD127^−^FOXP3^+^ Tregs. In healthy donors, these Tregs tended to produce more anti-inflammatory IL-10 and they produced less pro-inflammatory IFN-γ, while at the same time significantly downregulating LAG-3 expression in the presence of IMU-935 compared to controls. Using T cells from T1D donors, the expression of different markers had the same trend with a significant increase of IL-10 produced by Tregs when they were induced in presence of IMU-935 compared to the control. These results suggest an enhanced suppressive function of the IMU-935-induced CD25^+^CD127^−^FOXP3^+^ Tregs compared to control (Fig. S2F–H). In accordance with enhanced Treg induction, IMU-935 significantly reduced the T cell proliferation marker Ki67 (Figure 1G–J). In summary, these findings demonstrate that IMU-935 reduces human T cell activation and enhances Treg induction *in vitro*, particularly in conditions of increased immune activation.

### 2.2 IMU-935 enhances murine Treg induction *in vitro* under conditions of strong immune activation such as islet autoimmunity

**Figure 2:**
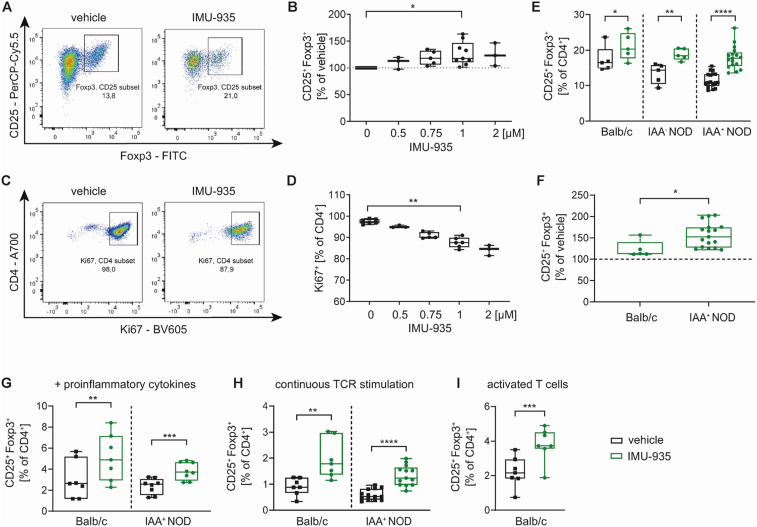
**IMU-935 enhances murine Treg induction *in vitro* during conditions of increased immune activation.** (**A, B**) Murine Treg induction assays *in vitro* using subimmunogenic TCR stimulation and naïve T cells from LNs of Balb/c mice in presence or absence of IMU-935. (**A**) Representative FACS staining using 1 μM IMU-935 and (**B**) summary plots of the Treg induction *in vitro* in presence of increasing concentrations of IMU-935, n = 3–9. (**C, D**) (**C**) Representative Ki67 staining after Treg induction *in vitro* using naïve T cells from Balb/c mice in presence or absence of 1 μM IMU-935. (**D**) Summary plots for Ki67^+^ T cell frequency after Treg induction *in vitro* in presence of increasing concentrations of IMU-935, n = 3–6. (**E**) Evaluation of Treg induction assays *in vitro* using subimmunogenic TCR stimulation and naïve T cells from pancreatic lymph nodes (pLN) of non-autoimmune prone Balb/c mice and NOD mice with different stages of autoimmunity presence (IAA^−^ vs. IAA^+^) in presence of 1 μM IMU-935 or the vehicle control, Balb/c n = 5, IAA^−^ NOD n = 5, IAA^+^ NOD n = 17. (**F**) Comparison of the effect of IMU-935 on Treg induction using T cells from Balb/c or IAA^+^ NOD mice from E represented as % of vehicle control. (**G-I**) Murine *in vitro* Treg induction during conditions of increased immune activation in presence of 1 μM IMU-935 or the vehicle control using naïve T cells isolated from LNs of Foxp3^GFP^ Balb/c mice or NOD mice. Treg frequencies in Treg induction using (**G**) subimmunogenic TCR stimulation in presence of pro-inflammatory cytokines IL-6, IFN-γ and IL-1β, Foxp3^GFP^ Balb/c n = 7, NOD n = 8, (**H**) continuous TCR stimulation, Foxp3^GFP^ Balb/c n = 7, NOD n = 13, or (**I**) Treg induction using activated T cells FACS-sorted as CD4^+^CD25^+^CD44^high^GFP^−^ to avoid contaminating Foxp3^+^ activated T cells, Foxp3^GFP^ Balb/c n = 7. Each data point represents one subject, experiments were performed in 2–3 technical replicates. (B, D) One-way ANOVA with Tukey's post hoc test for multiple comparisons, (E, F, G-I) Student's t test ∗ = p < 0.05; ∗∗ = p < 0.01; ∗∗∗∗ = p < 0.0001.

Next, we analyzed the effect of IMU-935 on murine Treg induction *in vitro*. In titration experiments using subimmunogenic TCR stimulation of naïve CD4^+^CD25^−^CD44^low^ T cells from non-autoimmune prone Balb/c mice, IMU-935 significantly improved Treg induction (Figure 2A,B, Fig. S3, A and B). This was accompanied by a reduction of the T cell proliferation marker Ki67 (Figure 2C,D). These findings are in line with the above mentioned human Treg induction data and with previous findings showing most efficient Treg induction in settings of limited cellular proliferation [29]. Similarly, IMU-935 increased Treg induction using naïve T cells from prediabetic NOD mice with or without ongoing islet autoimmunity (indicated by the absence/presence of insulin autoantibodies (IAA)) (Figure 2E). Importantly, IMU-935 was even more effective in inducing Tregs under subimmunogenic conditions *in vitro* when T cells were derived from settings of aberrant immune activation i.e., as observed during islet autoimmunity (positive effect of IMU-935, Balb/c: 123.25 % of vehicle; NOD with islet autoantibodies IAA+: 156.25 % of vehicle) (Figure 2F).

Additionally, we mimicked increased immune activation *in vitro*, as before in the human setting. IMU-935 significantly improved Balb/c Treg induction in all three experimental conditions of aberrant immune activation (Figure 2, G-I, Fig. S3C). Moreover, even when using T cells from NOD mice with ongoing islet autoimmunity in settings of strong immune activation, IMU-935 significantly enhanced Treg induction (Figure 2G,H). Overall, these data suggest that IMU-935 most efficiently improved Treg induction *in vitro* under conditions of increased immune activation including islet autoimmunity, a scenario that is particularly important for future translational efforts.

### 2.3 IMU-935 enhances RORγt expression in T cells and Tregs during *in vitro* Treg induction

**Figure 3:**
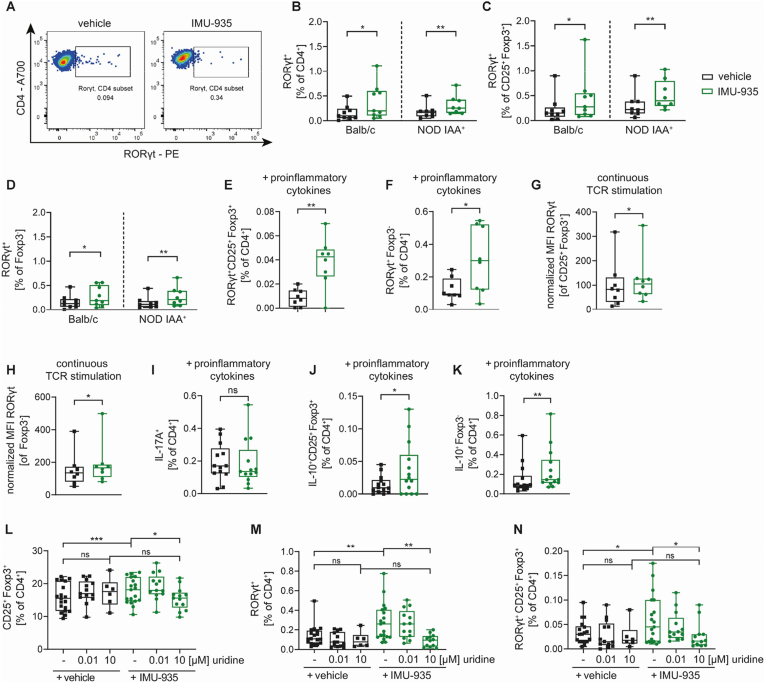
**IMU-935 induces upregulation of RORγt in T cells and Tregs in murine Treg induction *in vitro*.** (**A-D**) Murine Treg induction *in vitro* in presence of 1 μM IMU-935 or the vehicle control using limited TCR stimulation of naïve CD4^+^ T cells isolated from LNs of Balb/c or IAA^+^ NOD mice. (**A**) Staining examples for RORγt pregated on live CD4^+^ T cells. Evaluation of RORγt expression in (**B**) live CD4^+^ T cells (**C**) CD25^+^Foxp3^+^ Tregs or (**D**) Foxp3^-^ T cells, Balb/c n = 9, IAA^+^ NOD n = 8. (**E-H**) Analysis of RORγt expressing after Treg induction *in vitro* using naïve CD4^+^ T cells isolated from IAA^+^ NOD mice under the indicated conditions of increased immune activation in presence of 1 μM IMU-935 or the vehicle control. Frequencies of (**E**) RORγt^+^CD25^+^Foxp3^+^ Tregs or (**F**) Foxp3^−^CD4^+^ T cells, n = 7. Median fluorescence intensity (MFI) of RORγt in (**G**) CD25^+^Foxp3^+^ Tregs or (**H**) Foxp3^-^ CD4^+^ T cells, which was normalized to the respective isotype control, n = 8. (**I–K**) (**I**) Expression of IL-17A in CD4^+^ T cells (n = 13) and frequencies of (**J**) IL-10^+^CD25^+^Foxp3^+^ Tregs and (**K**) IL-10^+^Foxp3^−^CD4^+^ T cells after Treg induction *in vitro* in the presence of pro-inflammatory cytokines and 1 μM IMU-935 or the respective vehicle control using naïve T cells isolated from LNs of NOD mice, n = 14. (**L-N**) Subimmunogenic Treg induction *in vitro* using naïve T cells isolated from LNs of NOD mice in presence of 1 μM IMU-935 or the respective vehicle control and increasing concentrations of uridine. Evaluation of frequencies of (**L**) CD25^+^Foxp3^+^ Tregs, (**M**) RORγt^+^CD4^+^ T cells and (**N**) RORγt^+^CD25^+^Foxp3^+^ Tregs, n = 6–19. Each data point represents one subject, experiments were performed in 2–3 technical replicates. (B–K) Student's t test, (L–M) One-way ANOVA with Tukey's post hoc test for multiple comparisons ∗ = p < 0.05; ∗∗ = p < 0.01, ∗∗∗ = p < 0.001.

IMU-935 not only inhibits DHODH but also acts as a RORγt inverse agonist. Furthermore, RORγt and Foxp3 are both upregulated in Treg or T_H_17-promoting conditions *in vitro* [22]. This prompted us to examine RORγt expression following Treg induction *in vitro*. IMU-935 increased RORγt expression in subimmunogenic Treg induction in CD4^+^ T cells and induced Tregs from Balb/c and IAA^+^ NOD mice (Figure 3A–C). Similarly, RORγt expression was increased in Foxp3^-^ T cells (Figure 3D). This increased RORγt expression was validated using T cells from RORγt^GFP^Foxp3^RFP^ double reporter mice (Fig. S4, A and B). Likewise, IMU-935 enhanced frequencies of RORγt^+^ Tregs and RORγt^+^ Foxp3^-^ T cells in Treg induction under challenging conditions and using T cells from IAA^+^ NOD mice (Figure 3E–H). Increased expression of the *Rorc* gene that encodes for RORγt after continuous Treg induction *in vitro* in presence of IMU-935 was confirmed by qPCR analysis of CD4^+^ T cells (Fig. S4C).

Conceptually, RORγt expression is associated with the differentiation of pro-inflammatory IL-17A producing T_H_17 cells [30]. However, IMU-935 did not increase IL-17A expression in subimmunogenic Treg induction or Treg induction under challenging conditions (Figure 3I, Fig. S4, D and E). In contrast, IMU-935 significantly increased frequencies of anti-inflammatory IL-10^+^CD25^+^Foxp3^+^ Tregs (Figure 3J, Fig. S4F). IL-10 production was likewise increased in Foxp3^-^ T cells and total CD4^+^ T cells after Treg induction *in vitro* using challenging conditions or subimmunogenic TCR stimulation in presence of IMU-935 (Figure 3K, Fig. S4, G and H). In summary, the results show that IMU-935 fosters an anti-inflammatory phenotype in both *in vitro* induced Tregs and Foxp3^-^ T cells.

### 2.4 DHODH inhibition mediates the Treg-fostering effect of IMU-935

The results so far raised the question whether the Treg-fostering impact of IMU-935 results from its RORγt inverse agonism or from DHODH inhibition. To approach this experimentally, we used uridine supplementation during Treg induction. Uridine circumvents DHODH inhibition by supplying an exogenous source for pyrimidine synthesis, turning IMU-935 into a RORγt inverse agonist.

High concentrations of uridine significantly decreased the positive effect of IMU-935 on subimmunogenic Treg induction (Figure 3L). Likewise, proliferative capacity was restored in presence of uridine as evidenced by Ki67 staining (Fig. S5A). In addition, the increase in RORγt^+^ T cells and Tregs in response to IMU-935 was abrogated upon addition of high concentrations of uridine (Figure 3, M, N). In line with these results, uridine supplementation in Treg induction assays in presence of pro-inflammatory cytokines reduced the impact of IMU-935 on Treg induction and RORγt expression in Tregs and CD4^+^ T cells (Fig. S5, B-D).

To exclude the possibility of high concentrations of uridine affecting RORγt inverse agonism, we conducted experiments using the RORγt inverse agonist cedirogant. While cedirogant increased Treg induction capacity *in vitro*, it did not change RORγt expression in CD4^+^ T cells (Fig. S5, E and F). Supplementation of uridine did not affect Foxp3 or RORγt expression in presence of cedirogant or the vehicle control (Fig. S5, E and F). This indicates that uridine alone has no impact on Treg induction *in vitro* or on RORγt inverse agonism. Taken together, these results demonstrate that inhibition of DHODH is the driver of the Treg induction-enhancing effect of IMU-935.

### 2.5 DHODH inhibition by IMU-838 fosters murine and human Treg induction *in vitro* in settings of strong immune activation

**Figure 4:**
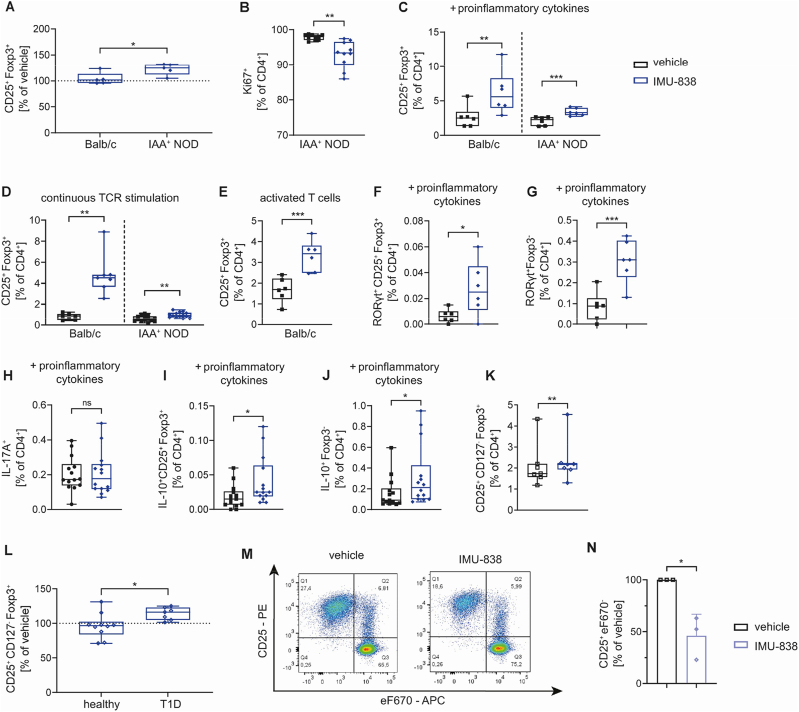
**DHODH inhibition by IMU-838 results in enhanced Treg induction during conditions of increased immune activation.** (**A**) Murine Treg induction *in vitro* using subimmunogenic TCR stimulation and naïve T cells from LNs of IAA ^+^ NOD and Balb/c mice. Comparison of the effect of 50 μM IMU-838 on Treg induction using T cells from Balb/c or IAA^+^ NOD mice represented as % of vehicle control, Balb/c n = 5, NOD n = 5, 5 independent experiments, one Balb/c and one NOD for comparison in the same experiment. (**B**) Summary plot of Ki67^+^ T cell frequencies after subimmunogenic Treg induction in presence of 50 μM IMU-838 or the vehicle control using T cells isolated from LNs of IAA^+^ NOD mice, n = 10. (**C-E**) Treg induction *in vitro* under conditions of increased immune activation in presence of 50 μM IMU-838 or vehicle control using T cells isolated from LNs of Foxp3^GFP^ Balb/c or IAA^+^ NOD mice. Treg frequencies in Treg induction using (**C**) subimmunogenic TCR stimulation in presence of pro-inflammatory cytokines IL-6, IFN-γ and IL-1β, Foxp3^GFP^ Balb/c n = 6, NOD IAA^+^ n = 6, and (**D**) continuous TCR stimulation, Foxp3^GFP^ Balb/c n = 7, NOD IAA^+^ n = 14. (**E**) Tregs were induced from activated T cells sorted as CD4^+^CD25^−^CD44^high^GFP^−^ to avoid contaminating Foxp3^+^ activated T cells, Foxp3^GFP^ Balb/c n = 6. (**F, G**) Frequency of (**F**) RORγt^+^CD25^+^Foxp3^+^ Tregs and (**G**) RORγt^+^Foxp3^−^CD4^+^ T cells in Treg induction assays *in vitro* using limited TCR stimulation and naïve T cells isolated from NOD mice in presence of pro-inflammatory cytokines and 50 μM IMU-838 or the vehicle control, n = 6. (**H-J**) (**H**) Expression of IL-17A in CD4^+^ T cells and frequencies of (**I**) IL-10^+^CD25^+^Foxp3^+^ Tregs and (**J**) IL-10^+^Foxp3^−^CD4^+^ T cells after *in vitro* Treg induction in the presence of pro-inflammatory cytokines and 50 μM IMU-838 or the vehicle control, n = 13. (**K, L**) Human subimmunogenic Treg induction using naïve T cells from PBMCs. (**K**) Frequencies of induced Tregs in presence of a vehicle control of 201 nM IMU-838 using T cells from blood of T1D donors. (**L**) Comparison of the effect of IMU-838 on Treg induction using T cells from healthy or T1D donors represented as % of vehicle control, healthy n = 10, T1D n = 7. (**M, N**) Human T cell proliferation assay to assess the CD4^+^ T cell response to Influenza (flu) vaccine using PBMCs from healthy blood donors. (**M**) Representative FACS staining of the proliferation of CD4^+^ T cells in presence of 5 μM IMU-838 or the respective vehicle control. (**N**) Summary plots of proliferating CD25^+^CD4^+^ T cells normalized to the vehicle control, n = 3. Each data point represents one subject, experiments were performed in 2–3 technical replicates. Student's t test, ∗ = p < 0.05; ∗∗ = p < 0.01; ∗∗∗ = p < 0.001.

Next, we assessed the impact of DHODH inhibition on Treg induction *in vitro* using IMU-838, a DHODH inhibitor, which does not inhibit RORγt. Activated T cells depend on DHODH and therefore are most susceptible to its inhibition. Thus, we reasoned that IMU-838 might be more efficient in settings of increased immune activation. Indeed, IMU-838 was superior in enhancing subimmunogenic Treg induction *in vitro* using T cells from settings of aberrant immune activation as observed during islet autoimmunity (Figure 4A). Increased Treg induction *in vitro* was accompanied by a reduction of the proliferation marker Ki67 (Figure 4B). In addition, IMU-838 improved Treg induction using naïve or activated CD4^+^ T cells from non-autoimmune prone Foxp3^GFP^ Balb/c mice or IAA^+^ NOD mice in settings of increased immune activation (Figure 4, C-E).

As shown above, IMU-935 relies on DHODH inhibition to affect RORγt expression, therefore the impact of IMU-838 mediated DHODH inhibition on RORγt expression was assessed. IMU-838 increased frequencies of RORγt^+^ Tregs as well as RORγt^+^ Foxp3^-^ T cells during subimmunogenic Treg induction *in vitro* in presence or absence of proinflammatory cytokines (Figure 4F,G, Fig. S6, A and B). The increased RORγt expression was again validated using T cells from RORγt^GFP^Foxp3^RFP^ double reporter mice (Fig. S6, C and D). In Treg induction assays using continuous TCR stimulation IMU-838 increased isotype control-normalized median fluorescence intensity (MFI) of RORγt in total CD4^+^ T cells and Foxp3^-^ T cells (Fig. S6, E and F). No change was observed in Tregs or in *Rorc* expression in T cells after continuous Treg induction (Fig. S6, G and H). A pro-inflammatory phenotype of induced RORγt^+^ Foxp3^-^ T cells was excluded by analyzing IL-17A vs. IL-10 cytokine profiles in these cells. While IL-17A expression was not changed by IMU-838 (Figure 4H), it increased frequencies of IL-10^+^CD25^+^Foxp3^+^ Tregs and IL-10^+^Foxp3^-^ T cells (Figure 4I,J). As for IMU-935, these results underline an anti-inflammatory phenotype of Tregs induced in presence of IMU-838.

As a proof-of-concept, we also tested the impact of an excess of uridine on IMU-838 function in Treg induction *in vitro*. The IMU-838-mediated increase in Treg frequencies as well as RORγt^+^CD4^+^ T cells and Tregs was reduced in presence of high concentrations of uridine (Figure S7, A-C). Likewise, T cell proliferation was partially restored (Fig. S7D). Next, we examined whether the impact on RORγt expression is a characteristic of the next-generation DHODH inhibitor IMU-838. Teriflunomide (Tef) was able to significantly enhance Treg induction as well as frequencies of RORγt^+^ T cells and RORγt^+^Foxp3^-^ T cells *in vitro* under immune-activating conditions (Fig. S7, E-H). However, its effect was significantly less pronounced compared to IMU-838. In addition, IMU-838 improved frequencies of RORγt^+^CD25^+^Foxp3^+^ Tregs better than Tef (Fig. S7H). Overall, these results show that DHODH inhibition improved Treg induction under immune-activating conditions. Importantly, IMU-838 was superior in enhancing Treg induction compared to the commonly used DHODH inhibitor Tef.

In addition, IMU-838 improved human subimmunogenic Treg induction *in vitro* using T cells isolated from peripheral blood of T1D patients (Figure 4K). Importantly, the effect of IMU-838 on Treg induction was significantly higher for T cells from T1D patients compared to control subjects (Figure 4L) which is in line with our murine results. Similarly, DHODH inhibition by IMU-838 reduced CD4^+^ T cell responses to flu antigens (Figure 4,M,N). In addition, naïve or memory CD4^+^ T cells stimulated with the super-antigen SEB proliferated less in presence of IMU-838 (Fig. S7, I-K). These findings indicate that IMU-838 limits CD4^+^ T cell activation which opens a broader window of opportunity to foster Tregs.

### 2.6 IMU-838 enhances Treg frequencies in NOD mice

**Figure 5:**
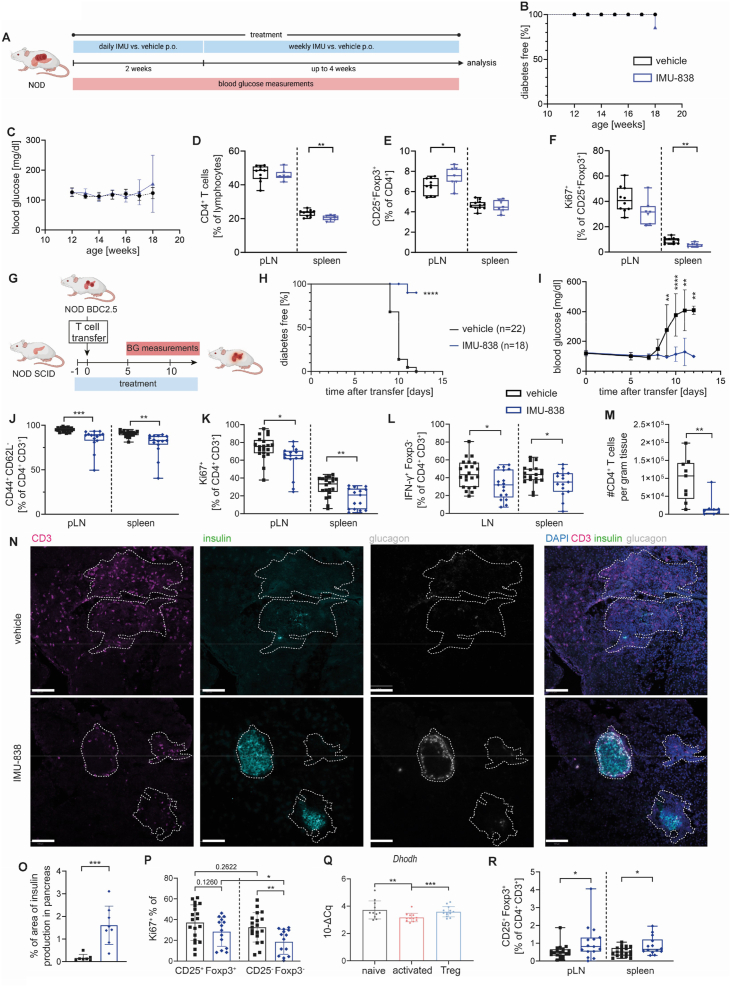
**IMU-838 fostered Tregs in two mouse model for T1D.** (**A**) Schematic representation of experimental design: 12-week-old NOD mice were treated daily with 150 mg/kg IMU-838 or PEG-400 as vehicle control for 14 days. After this, they were treated weekly for up to 4 weeks with IMU-838 or PEG-400 as vehicle control. The mice were monitored for hyperglycemia and analyzed at 18 weeks of age. (**B, C**) (**B**) Diabetes incidence and (**C**) mean blood glucose levels of vehicle or IMU-838 treated mice, vehicle n = 11, IMU-838 n = 7. (**D-F**) (**D**) Frequencies of CD4^+^CD3^+^ T cells, (**E**) CD25^+^Foxp3^+^ Tregs and (**F**) Ki67^+^CD4^+^CD3^+^ in the pLN (vehicle n = 11, IMU-838 n = 7) and spleen (vehicle n = 11, IMU-838 n = 7). (**G**) Schematic representation of experimental design: Immunodeficient NOD SCID mice were reconstituted with FACS-sorted naïve CD4^+^CD62L^+^TCRVb4^+^CD25^-^ T cells from NOD BDC2.5 mice and treated with IMU-838 or PEG-400 as vehicle control. The mice were monitored for hyperglycemia and analyzed between 10 and 12 d after transfer when the vehicle controls became diabetic. (**H, I**) (**H**) Diabetes incidence and (**I**) mean blood glucose levels of vehicle or IMU-838 treated mice, vehicle n = 22, IMU-838 n = 18. (**J-L**) (**J**) Frequencies of CD44^+^CD62L^−^CD4^+^CD3^+^ T cells, (**K**) Ki67^+^CD4^+^CD3^+^ in the pLN (vehicle n = 20, IMU-838 n = 15) and spleen (vehicle n = 20, IMU-838 n = 15) and (**L**) IFNγ-producing Foxp3^−^CD4^+^CD3^+^ T cells in the LNs (vehicle = 22, IMU-838 n = 17) and spleen (vehicle n = 19, IMU-838 n = 15). (**M**) Number of infiltrating CD4^+^ T cells in the pancreas per gram tissue, vehicle n = 9, IMU-838 n = 8. (**N**) Representative immune fluorescence staining of pancreatic tissue sections stained for CD3 (magenta), insulin (green), glucagon (white) and nuclei/DAPI (blue). The scale bar is 100 μm. (**O**) Quantification of the percentage of the area of insulin producing cells in the pancreatic area of the tissue section, vehicle n = 7, IMU-838 n = 8. (**P**) Frequencies of Ki67^+^ cells of CD25^+^Foxp3^+^ Tregs or CD25^-^Foxp3^-^ non-Treg cells in the spleen, vehicle = 19, IMU-838 n = 14. (**Q**) qPCR analysis of *Dhodh* gene expression of FACS-sorted CD44^low^CD25^−^CD4^+^ (naïve, n = 11), CD44^high^CD25^−^CD4^+^ (activated, n = 12) T cells or CD25^+^CD4^+^ (Tregs, n = 13) isolated from LNs of NOD mice. Gene expression was normalized to Histone H3. (**R**) Frequencies of CD25^+^Foxp3^+^ Tregs in pLN (vehicle n = 20, IMU-838 n = 15) and spleen (vehicle n = 19, IMU-838 n = 14) of IMU-838 or vehicle treated mice. G-L, P, R: seven, M: two and O: three independent experiments, each data point represents one subject. (B, H) Log-rank (Mantel–Cox) test, (C, I) Two-way ANOVA with Sidak's multiple comparisons test, (D-F, J-P, R) Student's t test (Q) One-way ANOVA with Tukey's post hoc test for multiple comparisons. ∗ = p < 0.05; ∗∗ = p < 0.01; ∗∗∗ = p < 0.001; ∗∗∗∗ = p < 0.0001.

Based on the positive impact of IMU-838 on Treg induction in *vitro*, first we evaluated the Treg-fostering potential of IMU-838 in a classical T1D model *in vivo*. Specifically, in this first set of experiments, we focused on a possible benefit of IMU-838 during early stages of islet autoimmunity. For this, twelve-week-old NOD mice were treated daily with 150 mg/kg IMU-838 or vehicle (PEG-400) for 14 consecutive days, followed by once-weekly dosing for an additional four weeks, for a total of a six-week study period (Figure 5A). By the end of the study, only one IMU-838–treated mouse developed diabetes (Figure 5B); however, no significant differences in mean blood glucose levels were observed between the treatment and control groups (Figure 5C). Flow cytometric analysis of pancreatic lymph nodes (pLN) and spleen revealed a significant reduction in CD4^+^ T cells in the spleens of IMU-838-treated mice (Figure 5D). Notably, IMU-838 treatment significantly increased the frequency of CD25^+^Foxp3^+^ Tregs in the pLN (Figure 5E) and reduced CD4^+^ T cell proliferation in both the pLN and spleen, suggesting an immunomodulatory effect that may contribute to disease protection (Figure 5F).

### 2.7 DHODH inhibition by IMU-838 delays disease progression in a model of accelerated T1D

Importantly, our *in vitro* data had indicated that DHODH inhibition is most efficient in enhancing Treg induction in conditions of strong aberrant immune activation. Thus, we argue that IMU-838 has maximum potential to interfere with aberrant immune activation and disease progression in settings of an aggressive disease phenotype. To test this hypothesis, IMU-838 was applied to a model of accelerated T1D induced by adoptive T cell transfer [34]. Diabetogenic naïve TCRVβ4^+^CD4^+^ T cells were isolated from prediabetic NOD BDC2.5 mice (Fig. S8A). To this end, immunodeficient NOD SCID mice were reconstituted with these diabetogenic T cells, treated with IMU-838 or vehicle control and monitored for elevated blood glucose (Figure 5G). Control mice became diabetic within 9–12 days after T cell transfer (i.e. 22/22 mice) while IMU-838 application largely protected the mice from T1D development by that time point (i.e. 1/18 mice) (Figure 5H). The protective effect of IMU-838 was further supported by a significant reduction in mean blood glucose levels in IMU-838-treated mice compared to control animals (Figure 5I). Mice were sacrificed when vehicle treated mice of the respective cohort became diabetic. IMU-838 treated mice showed a significant reduction in CD4^+^ T cell activation and proliferation in pLN and spleen (Figure 5J,K, Fig. S8B). Likewise, IFN-γ production of Foxp3^−^CD4^+^ T cells in spleen and LNs was decreased (Figure 5L). While the activation and proliferation of the pancreas-residing T cells did not change (Figure S9, A‒C), IMU-838 treatment significantly reduced the number of total CD4^+^ T cells in the pancreas (Figure 5M). In case of a longer study, mice were killed maximum two days after becoming diabetic, and IMU-838 treated mice showed a delay in T1D development, and a slightly lower mean blood glucose (Figure S9, D and E). Here, IMU-838 treatment led to an increase in CD25^+^Foxp3^+^ Tregs in the pancreas, with a tendency towards a decreased T cell proliferation compared to vehicle-treated mice (Figure S9, F and G). In the pLN and spleen, IMU-838-treated mice also showed an increased frequency of CD25^+^Foxp3^+^ Tregs together with a decreased T cell proliferation as shown in the shorter time point (Figure S9, H and I).

Moreover, T cell infiltration in the pancreas and residual insulin production was also assessed by immunofluorescence. While control mice showed a widespread CD3 infiltration of islets and pancreas, T cell infiltration in IMU-838 treated mice was lower and largely restricted to the islets (Figure 5N). Quantification revealed a trend towards reduced CD3 mean intensity in the whole pancreas (Fig. S9J). Furthermore, islets with residual insulin production in pancreata of IMU-838 treated mice tended to have a reduced mean CD3 intensity (Fig. S9K). This suggests that insulin-producing islets are less infiltrated with CD4^+^ T cells in IMU-838 treated mice. The remaining pancreatic insulin production of IMU-838 treated mice was higher compared to control mice. We observed a significantly larger area of insulin producing cells as well as a higher average insulin intensity of individual islets in pancreata of IMU-838 treated animals (Figure 5O).

### 2.8 Pyrimidine restriction by IMU-838 fosters Tregs in a model of accelerated T1D

IMU-838 treatment reduced the proliferation of CD4^+^ T cells. Next, we assessed whether IMU-838 affected the proliferation of CD25^+^Foxp3^+^ Tregs and CD25^-^Foxp3^-^ non-Tregs differentially. Interestingly, frequencies of Ki67^+^CD25^+^Foxp3^+^ Tregs showed no significant reduction in splenocytes of treated mice while the proliferation of non-Tregs dropped significantly in mice receiving IMU-838 (Figure 5P). This corresponded to a drop in Ki67 frequency of 26% in Tregs or 47% in Foxp3^-^ T cells upon IMU-838 treatment. This finding could hint towards a lower sensitivity of Tregs to DHODH inhibition compared to activated T cells/non-Tregs. Confirming our *in vivo* results, in Treg induction assays *in vitro* IMU-838 affected proliferation of induced Tregs to a lower extent compared to non-Tregs (Fig. S9L). We next analyzed the *Dhodh* mRNA expression in various CD4^+^ T cell subsets of NOD mice and observed significantly reduced *Dhodh* expression in activated T cells compared to naïve or CD25^+^Foxp3^+^ Tregs (Figure 5Q). This could hint towards a higher capacity of Tregs to produce pyrimidines which in turn makes them less vulnerable to DHODH inhibition.

Given the strong anti-proliferative effect of IMU-838, cell numbers of CD25^-^Foxp3^-^ non-Tregs were drastically reduced in pLN of treated mice (Fig. S9M). In contrast, Tregs were spared from this effect resulting in equal Treg cell numbers in pLN (Fig. S9N). Accordingly, frequencies of CD25^+^Foxp3^+^ Tregs were increased in pLN and spleen of IMU-838 treated mice (Figure 5R). Foxp3 staining controls of transferred cells revealed a small percentage of transferred CD25^-^Foxp3^+^ Tregs (Fig. S9O). This indicates that Tregs can either be stabilized, expanded, or induced after T cell transfer. Importantly, Tregs from treated mice expressed similar frequencies of the Treg suppression markers CTLA4 and PD-1 compared to control mice suggesting that they are equally suppressive (Fig. S9, P and Q).

Overall, this shows that inhibition of DHODH using IMU-838 in a model of accelerated T1D was able to reduce T cell activation and proliferation. The findings support the concept that Tregs are spared from the anti-proliferative effect of DHODH inhibition, thereby resulting in the fostering of Tregs, most probably in an indirect fashion. In addition, reduced immune cell infiltration into the pancreas and delayed T1D development was confirmed in two independent mouse models.

### 2.9 DHODH inhibition by IMU-838 reduces incidences of T1D in the RIP-LCMV-GP mouse model

**Figure 6:**
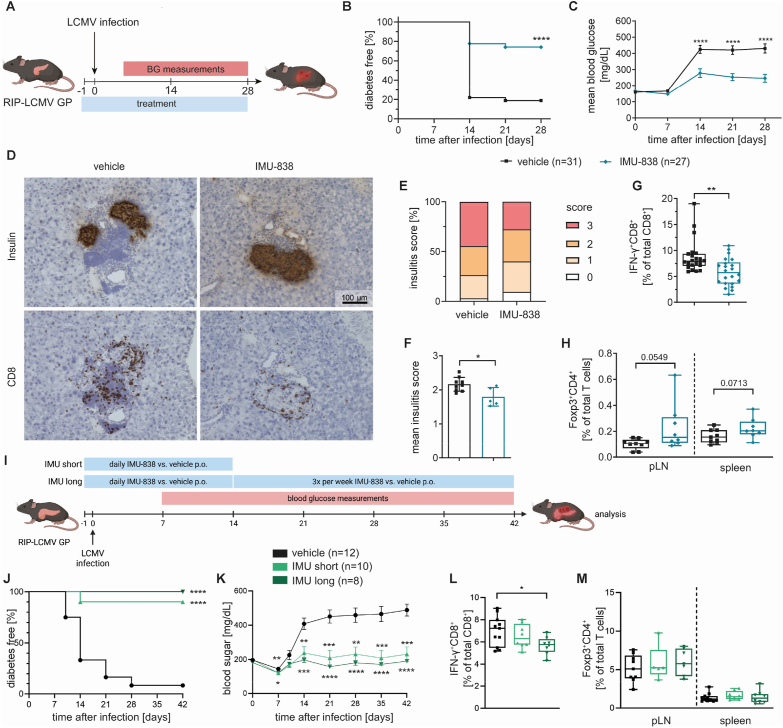
**IMU-838 reduces incidences of T1D in the RIP-LCMV-GP mouse model both in a short-term and in long-term study.** (**A**) Schematic representation of experimental design: RIP-LCMV-GP transgenic mice were infected with LCMV and treated with IMU-838 or a respective vehicle control. Mice were monitored for high blood glucose and analyzed at d14 or d28. (**B, C**) (**B**) Diabetes incidence and (**C**) the mean blood glucose levels of vehicle or IMU-838 treated mice, vehicle n = 31, IMU-838 n = 27 (**D-F**) Insulin, CD8 and hematoxylin-staining of pancreas cryosections. (**D**) Representative images stained cryosections of pancreata from vehicle and IMU-838 treated mice. (**E**) Grading of insulitis from IMU-838 treated mice as described in Fig. S10A at the end of the experiment. (**F**) Summary of the mean insulitis score, vehicle n = 9, IMU-838 n = 5. (**G**) GP33-specific CD8^+^ T cells in the spleen at d28 of the experiment, vehicle n = 22, IMU-838 n = 22. (**H**) Analysis of Foxp3^+^CD4^+^ T cells in the pLN and spleen at d14 of the experiment, vehicle n = 9, IMU-838 n = 8. (**I**) Schematic representation of experimental design: RIP-LCMV-GP transgenic mice were infected with LCMV and treated with IMU-838 or a respective vehicle control daily for 14 days. The IMU-838 treated mice were then split in subgroups: one group (IMU short) did not receive further treatment and another one (IMU long) received IMU-838 three times a week until the end of the study. (**J, K**) (**J**) Diabetes incidence and (**K**) mean blood glucose levels of vehicle, IMU-838 short and IMU-838 long treated mice, vehicle n = 12, IMU short n = 10, IMU long n = 8. (**L**) IFNγ^+^CD8^+^ T cells in the spleen at d42 of the experiment, vehicle n = 12, IMU-838 short n = 10, IMU-838 long n = 8. (**M**) Analysis of Foxp3^+^CD4^+^ T cells in the pLN and spleen at d42 of the experiment, vehicle n = 12, IMU-838 short n = 10, IMU-838 long n = 8. (A–H) Experiments were performed in two or more independent experiments, each data point represents one subject. (B, J) Log-rank (Mantel–Cox) test, (C, K) Two-way ANOVA with Sidak's multiple comparisons test, (G, F, L, M) Student's t test. ∗ = p < 0.05; ∗∗ = p < 0.01; ∗∗∗ = p < 0.001; ∗∗∗∗ = p < 0.0001.

To validate our findings that DHODH inhibition is most efficient in settings of aberrant immune activation, in the next step we chose a virus-induced T1D mouse model as an independent experimental setup performed in a different laboratory. Specifically, the goal was now to confirm the potential of IMU-838 to reduce the progression to T1D *in vivo*. To that end, we employed the RIP-LCMV-GP mouse model, which has been used to study CD8^+^ T cell responses [31]. In this model, transgenic mice express the glycoprotein (GP) of the lymphocytic choriomeningitis virus (LCMV) under control of the rat insulin promoter (RIP) in the β-cells of the pancreas [32,33]. RIP-LCMV-GP mice were infected with LCMV according to established protocols, treated with IMU-838 or with a vehicle control and monitored for high blood glucose levels (Figure 6A). 84% (i.e. 26/31 mice) of control mice developed T1D by day 28 (Figure 6B). In contrast, IMU-838 treatment significantly reduced T1D incidences and only 26% (i.e. 7/27 mice) of mice developed T1D within 28 days. Accordingly, the average blood glucose of IMU-838 treated mice was significantly reduced starting from day 14 after LCMV infection (Figure 6C). In addition, histopathological analysis showed reduced immune cell infiltration in the pancreas of IMU-838 treated mice as well as significantly reduced mean insulitis scores (Figure 6D–F, Fig. S10A). In this model, GP33-specific T cells mediate the destruction of the pancreatic beta cells [32,33]. In accordance with reduced beta cell destruction, we observed reduced frequencies of beta cell-specific IFN-γ-producing CD8^+^ T cells in the spleen of IMU-838 treated mice (Figure 6G, Fig. S10B). Foxp3^+^ Tregs tended to be increased in the pLN (*p* = 0.0549) and spleen (*p* = 0.0713) of IMU-838 treated compared to control mice which hints towards a beneficial impact of DHODH inhibition on Tregs *in vivo* (Figure 6H). Overall, these results strengthen the observations obtained in the NOD-related T1D models and confirm that DHODH inhibition by IMU-838 is able to reduce T1D incidences in the RIP-LCMV-GP mouse model.

To determine whether the reduced T1D incidence observed in the RIP-LCMV-GP mouse model was due to a delay in disease onset, or whether it indicated true protection from disease development with a sustained therapeutic benefit, we repeated the experiment using different settings. This also allowed us to assess whether the beneficial effects could be maintained even with reduced dosing of the drug. We treated RIP-LCMV-GP mice starting one day before the infection daily for 14 days either with vehicle or IMU-838. Afterwards, the IMU-838 treated mice were split in two groups: one received the treatment three times a week (IMU-long) and the second group was not treated further (IMU-short) (Figure 6I). Interestingly, in both treated groups T1D development was prevented. None of the IMU-long treated mice (0/8) and only one of the IMU-short (1/10) became diabetic compared to the vehicle treated mice (11/12 mice developed T1D) within 42 days after infection (Figure 6J). This is also shown by the mean blood glucose levels: both IMU-short and IMU-long treated groups have significantly lower levels compared to vehicle treated mice (Figure 6K). At day 42, spleen and pancreatic lymph nodes of these mice were analyzed via flow cytometry. Reduced frequencies of beta cell-specific IFN-γ-producing CD8^+^ T cells were visible in the spleen of IMU-838 treated mice, in particular the reduction was significant in the IMU-long treated mice (Figure 6L). At day 42, a trend to an increase of Foxp3^+^ Tregs was visible in the pancreatic lymph nodes of both IMU-838 treated groups compared to the vehicle treated (Figure 6M). Overall, these results show that DHODH inhibition by IMU-838 not only delays the onset of T1D but is able to protect most mice (90%) from developing the disease. This suggests a sustained therapeutic benefit in this model.

## 3 Discussion

The development of effective immunomodulatory therapies for T1D is hindered by increased T cell activation and Treg impairments. During islet autoimmunity, aberrant immune activation is a strong limiting factor for Treg-based immune modulation. To overcome this limitation, controlling overshooting immune activation could function as a relevant strategy to allow for efficient Treg targeting.

In this study, we identify DHODH inhibition, which restricts the availability of *de novo* synthesized pyrimidines, as a novel strategy to foster Tregs during islet autoimmunity and T1D. We provide evidence for several mechanisms by which DHODH inhibition can influence T cells and Tregs which likely act in concert. Firstly, T cell proliferation and activation is drastically reduced upon pyrimidine starvation which is beneficial for Treg induction [29]. We demonstrate a superior effect of DHODH inhibition during conditions of increased (auto-) immune activation. Here, we mimicked strong immune activation conditions *in vitro* by employing various approaches. Additionally, the two T1D mouse models which were used here are characterized by an increased immune activation and therefore fast onset of T1D. We employed a virus-induced T1D mouse model which has been used to study CD8^+^ T cell responses and a T1D transfer model in which transferred islet antigen-specific CD4^+^ T cells induce beta cell destruction in the pancreas [31,34]. Since under such conditions of overshooting immune activation efficient Treg induction and stability is limited, pyrimidine restriction opens a broader window of opportunity to foster Tregs [11,12]. *De novo* pyrimidine synthesis and DHODH activity are essential for activated and proliferating T cells [14]. This higher dependence on *de novo* synthesised pyrimidines might explain the stronger Treg-fostering effect in states of increased immune activation.

As a second Treg-fostering mechanism, our data indicate that Tregs are less affected by the anti-proliferative effect of DHODH inhibition compared to non-Treg. This suggest that Tregs are more resistant to a loss of pyrimidines. Evidence that pyrimidine starvation affects cell types differently comes also from human studies of Tef-treated patients with RRMS [35]. In addition, it has been recently shown that DHODH inhibition impacts effector precursor but not memory precursor CD8^+^ T cells [36]. This is due to a reduced proliferation rate of the latter which likely results in a lower per time demand of pyrimidines. Furthermore, effector committed CD8^+^ T cells have a lower expression of key enzymes of the pyrimidine *de novo* biosynthesis pathway which renders them more vulnerable to DHODH inhibition [36]. We also observed an increased *Dhodh* expression in Tregs compared to activated T cells which might suggest an advantage of Tregs to produce *de novo* pyrimidines. Future studies will be needed to further examine the pyrimidine demand of Tregs and the lower vulnerability of Tregs towards DHODH inhibition.

To investigate the impact of DHODH inhibition in fostering Tregs, we utilized two novel drug candidates, IMU-935 and IMU-838, both of which demonstrated potent reduction of T cell activation [15,21,25]. Importantly, the DHODH inhibitor IMU-838 was more potent in fostering Tregs *in vitro* when compared to the first-generation DHODH inhibitor Tef. This is in line with a previous study showing that IMU-838 inhibits human T cell proliferation with a 2.65 fold lower EC_50_ compared to Tef [27]. IMU-935 has a dual function as a RORγt inverse agonist and DHODH inhibitor. There is contradictory evidence whether inhibition of RORγt by inverse agonists enhances Treg induction [37,38]. We demonstrate that IMU-935 enhances Treg induction *in vitro* through its properties as DHODH inhibitor, rather than as RORγt inverse agonist. A beneficial impact of DHODH inhibition on Tregs was already observed in several other studies. Treatment of collagen-induced arthritis rats with the DHODH inhibitor Leflunomide resulted in increased Treg frequencies [39]. Likewise, the frequency of Helios^−^ Tregs was elevated in the blood of multiple sclerosis (MS) patients upon Tef treatment [35]. This is in line with the previously reported enhanced *in vitro* Treg induction capacity upon DHODH inhibition [40]. Despite these initial insights, in this study we demonstrate a Treg-fostering impact of DHODH inhibition in the context of islet autoimmunity and T1D, which was until now not well explored.

IMU-838 and IMU-935 enhance RORγt^+^ expression in Tregs and non-Tregs during Treg induction. Uridine supplementation experiments indicate that this effect depends on DHODH inhibition. Nonetheless, this did not coincide with a pro-inflammatory phenotype of IL-17 producing T_H_17 cells. We observe higher frequencies of IL-10 producing Foxp3^-^ T cells and Foxp3^+^ Tregs upon DHODH inhibition indicating a shift to an anti-inflammatory state. Likewise, several studies describe an anti-inflammatory fate of T_H_17 cells that is characterized by IL-10 production [41,42]. In line with these observations, DHODH inhibition reduced the production of IL-17 following *in vitro* PHA stimulation [27]. Similar to our results, the increase in RORγt expression was observed in Treg induction experiments using TGF-β [22,43]. In this study, we do not use TGF-β due to the instable phenotype of Tregs induced *in vitro* in its presence [44]. Still, we observe increased RORγt expression in Tregs and non-Tregs upon pyrimidine restriction. Mechanistically, Boardman et al. showed that a T_H_17 cell-targeting compound resulted in reduced IL-17 expression while *RORC2* expression was increased [45]. This could hint towards a feedback mechanism of increased RORγt expression due to repressed IL-17 expression. Another study suggests a compensatory upregulation of RORγt due to an increase in Foxp3 expression [46]. Future investigations into the functional capability of the induced RORγt^+^Foxp3^+^ Tregs upon DHODH inhibition will be key to address their biological relevance. A potential pathogenic contribution of T_H_17 cells to T1D development is still controversial [[47], [48], [49]]. It is currently assumed that RORγt^+^Foxp3^-^ T cells and RORγt^+^ Tregs have only a minor contribution to islet autoimmunity and progression to T1D.

To confirm a functional *in vivo* relevance for T1D, we investigated DHODH inhibition in three mouse models characterized by different degrees of immune activation: 1) a model characterized by mild immune activation and presence of early stages of islet autoimmunity (NOD mouse model), 2) one model defined by an aggressive phenotype (transfer model of accelerated T1D) on the NOD background and 3) one model with a fast and aggressive onset of T1D (RIP-LCMV-GP mouse model). Of note, DHODH inhibition resulted in reduced T1D incidence and improved key parameters used to evaluate T1D pathology in all three pre-clinical mouse models for T1D.

Our results are in line with a beneficial effect of DHODH inhibitors in other autoimmune diseases, such as MS, Lupus and colitis [26,27,50]. However, their use in ongoing islet autoimmune activation and T1D was until now insufficiently explored. First hints that DHODH inhibition might be particularly relevant for T1D treatment came from obesity and T2D studies. Zhang et al. showed that in a model of obesity-induced diabetes the DHODH inhibitor BAY2402234 improved glucose control and delayed beta cell loss [51]. This was linked to an increased expression of the cytokine GDF15, which itself protects from beta cell death and reduces incidences of T1D [52]. We employed three distinct T1D mouse models, establishing a gradient of disease kinetics, ranging from the relatively slow and mild progression seen in NOD mice to the rapid and aggressive onset observed in the adoptive transfer model of accelerated T1D. In NOD mice, which exhibit relatively mild immune activation, IMU-838 treatment did not lead to a detectable delay in T1D development — potentially due to the limited observation period. However, both reduced T cell proliferation and increased Treg induction were evident. To better mimic the intense immune activation required for DHODH inhibition to exert its full effects, we used a transfer model of accelerated T1D development. This model presents a highly aggressive phenotype with rapid onset of disease. In this setting, IMU-838 treatment delayed disease onset, although it did not fully prevent T1D development. Therefore, it is reasonable to assume that the main mechanism of action of DHODH inhibition is the reduction of T cell activation and proliferation. Beta cell death can be mediated by proinflammatory cytokines IL-1β, IFN-γ or TNF-α [53,54]. Thus, reduced inflammation and IFN-γ production due to DHODH inhibition likely also improves beta cell survival. However, we cannot exclude a minor direct effect of DHODH inhibition on the beta cells themselves. As a second rather indirect effect of pyrimidine starvation, the state of reduced immune activation also provides an environment that is beneficial for Treg induction, stability, and expansion. Therefore, in follow-up studies it would also be interesting to investigate an additional benefit of combinatorial therapy options of DHODH inhibition and agents that foster antigen-specific Tregs during ongoing islet autoimmune activation.

In the RIP-LCMV-GP mouse model we followed T1D development for up to four weeks and showed a significant delay of T1D development. We performed follow-up studies on diabetes development either with a longer continuous treatment regime or after stopping the treatment. Our data for continuous treatment for up to four weeks, however, hint towards a longer protection from T1D that is confirmed by the longer follow up. With respect to the effects after treatment stop, IMU-838 has a plasma half-life of about 30h in humans [27].

The T1D transfer mouse model makes use of diabetogenic T cells reactive to a chromogranin A related epitope [34]. However, insulin is the major driver of T1D in the NOD mouse model and an important autoantigen in human T1D [55,56]. Therefore, future studies using models with another autoantigen, or more than one autoantigen will be helpful to validate our findings. T1D development in the NOD mouse model is very heterogenous and stratification of mice according to their risk for developing symptomatic T1D is not possible. This fact combined with the mild immune activation that characterizes this mouse model makes it difficult to see differences on T1D development using IMU-838. Therefore, the decision to include more aggressive T1D models allows for a timed treatment regimen of mice that have a similarly high risk of developing T1D and will benefit from immediate treatment. The T1D transfer model is characterized by a highly aggressive immune activation environment, initiated by the transfer of a pool of naïve CD4^+^ T cells. In this setting, the induction of Tregs by DHODH inhibition is delayed, as it requires time to take effect, while the diabetogenic T cells rapidly initiate tissue damage. As a result, the observed delay in T1D onset in this model is limited and not sustained. In contrast, DHODH inhibition confers protection in the RIP-LCMV-GP mouse model, which features ongoing, systemic autoimmune activation. In this context, the compound can simultaneously reduce T cell proliferation and foster Treg induction, thereby effectively preventing the development of T1D.

Of therapeutic relevance, we show that DHODH inhibition can enhance human Treg induction *in vitro* in settings of increased autoimmune activation. Noteworthy, IMU-838 has an advanced clinical and safety profile and showed promising results in a phase 2 clinical trial in RRMS [17,27,57]. In T1D, as the beta cell mass declines throughout the disease, the autoimmune process also subsides. Based on our findings and the mechanism of action, DHODH inhibition by IMU-838 appears highly effective in conditions of strong (auto-) immune activation. This indicates an optimal window for DHODH inhibition in T1D during the pre-symptomatic phase or early stage 3. Initially, T1D treatment as tertiary intervention to preserve beta cell function would target recently diagnosed T1D patients.

Our findings suggest that DHODH inhibition could also be promising for a preventive, secondary intervention aimed at increasing the time before symptomatic disease onset. The pre-symptomatic phase exhibits significant heterogeneity and can vary drastically among individuals, underscoring the complexity of identifying individuals at increased risk of developing symptomatic T1D. Genetic risk scores are powerful and cost-effective tools to determine the risk for developing T1D. Bonifacio et al. merged the established Winkler and Oram scores, which included HLA and T1D susceptibility loci [58]. Using children enrolled in the Environmental Determinants of Diabetes in the Young (TEDDY) study, they showed that children with a merged genetic score >14.4 have a 7.6% risk to develop T1D by the age of 10 years compared to 2.7% in children with a score of ≤14.4 [58]. A recent study provided an improved risk score by combining a genetic risk score with the family history in first-degree relatives and the presence of autoantibodies [59,60]. Based on the established risk scores individuals can be stratified according to their chance to develop symptomatic T1D. Treatment of the individuals with the highest risk could decelerate beta cell destruction and increase the time before the onset of clinical symptoms due to dampened autoimmune activation.

Overall, we propose DHODH inhibition as a promising candidate for future therapeutic strategies with the goal to dampen autoimmune activation in islet autoimmunity and T1D.

## 4 Materials and methods

### 4.1 Human subjects

All human studies comply with the relevant ethical regulations for work with human participants and all study participants gave informed written consent prior to inclusion in the “B11 – Mechanismen der Immunaktivierung vs. Toleranz in Autoimmunem Typ 1 Diabetes” project (2019–510_4-S-SR). Fresh venous blood was collected using sodium heparin tubes and blood volumes were based on EU guidelines with a maximal blood volume of 2.4 mL per kg of body weight. Healthy (n = 15, 8 females, 7 males, median age 27.97) or T1D subjects (time from T1D onset >1 year, n = 12, 9 females, 3 males, median age 25.3) were included in the experiments. Subjects with ongoing or recent flu-like symptoms were excluded for experiments involving Treg induction *in vitro*.

For T cell proliferation assay, buffy coat or blood leucocyte cones samples were used. Buffy coat samples (Leukopaks) derived from peripheral blood of healthy individuals that donated erythrocytes for clinical use. These were purchased from the blood donation service DRK (Deutsche Rote Kreuz: Blutspendedienst Ost GmbH Dresden). Blood leucocyte cones from NHSBT platelet donation were obtained from London Tooting Blood Donor Centre (NHS Blood &Transplant).

### 4.2 Mice

NOD/ShiLtJ (NOD), Balb/c.Cg-Foxp3tm2Tch/J (Foxp3^GFP^ Balb/c), CBy.PL(B6)-*Thy1a*/ScrJ (Balb/c), NOD.Cg-*Prkdcscid*/J (NOD SCID), NOD.Cg-Tg TcraBDC2.5,TcrbBDC2.5)1Doi/DoiJ (NOD BDC2.5) were obtained from the Jackson Laboratory and maintained by in-house breeding. RIP-LCMV-GP transgenic mice (RIP-GP) were generated and screened by PCR as previously described [61,62]. RORγt^GFP^Foxp3^RFP^ double reporter mice have been described earlier [23]. NOD mice were stratified according to their IAA status at day 40 and 70 and the date of death. To obtain the insulin autoantibody status of NOD mice, a mouse high specificity/sensitivity competitive IAA assay in an ELISA format using plasma from NOD mice was performed as described earlier [11,63]. For experiments using NOD or NOD BDC2.5 mice blood sugar was measured using Accu-Check Aviva® glucometer.

BDC2.5 mice were genotyped by PCR using Dream Taq Green DNA Polymerase (Thermo Fischer Scientific) and the primer listed Table S1 according to the manufacturer's instructions. For analysis the PCR was run on a 2% (w/v) TAE agarose gel with Midori Green (Biozym).

Controls were age- and sex-matched mice. Whenever possible, littermates were used. Mice were randomly allocated to experimental group.

Mice were bred and maintained on a 12 h/12 h light dark cycle at 25 °C with ad libitum access to water and a standard diet (Altromin #1314) under specific pathogen free conditions at the animal facility of the Helmholtz Center Munich or the Goethe University Frankfurt, Germany, according to the Institutional Animal Committee Guidelines. Ethical approval for all mouse experimentations has been received by the District Government of Upper Bavaria, Munich, Germany (#ROB-55.2-2532.Vet_02-18-173, # ROB-55.2-2532.Vet_02-17-63, # ROB-55.2-2532.Vet_02-21-196 and # ROB-55.2-2532.Vet_02-23-116) or by the local Ethics Animal Review Board, Darmstadt, Germany (V54-19c20/15-FU/1170 and V54–19c20/15-FU/2061).

### 4.3 NOD mouse model

IMU-838 was dissolved in PEG-400 and PEG-400 alone served as vehicle control. 12-week-old NOD mice were treated with 150 mg/kg IMU-838 or the vehicle control by oral gavage daily for 14 days. After the first two weeks, oral gavage was performed once a week either with IMU-838 or with vehicle control. Body weight was monitored and in rare cases in which mice showed an interim trend of losing body weight, treatment was paused or dose-reduced and resumed after a maximum of 4 days. At that time body weight had stabilized and remained stable until the end of the treatment period. Blood glucose (BG) was measured from the tail vein. Mice with a measurement above 300 mg/dL were considered diabetic. The experiment was terminated after 6 weeks from the starting point.

### 4.4 Diabetes induction by adoptive transfer

To induce diabetes, diabetogenic T cells from TCR transgenic BDC2.5 NOD mice were transferred into NOD SCID mice as described earlier [34]. In brief, CD4^+^ T cells from pre-diabetic BDC2.5 NOD mice were enriched, and sort purified as described later. 1.4 Mio sorted live CD4^+^CD62L^−^ CD25^−^TCRVβ4^+^ T cells per mouse were transferred i.v. into NOD SCID mice. Post-sort purity controls were obtained by fixation and intracellular staining for Foxp3 using an aliquot of the sorted cells.

IMU-838 was dissolved in PEG-400 and PEG-400 alone served as vehicle control. Mice were treated daily with 150 mg/kg IMU-838 or the vehicle control by oral gavage starting the day before the transfer with half the dose. Blood glucose (BG) was measured from the tail vein. Mice with a measurement above 300 mg/dL were considered diabetic. Body weight was monitored and in rare cases in which mice showed an interim trend of losing body weight, treatment was paused or dose-reduced and resumed after a maximum of 4 days. At that time body weight had stabilized and remained stable until the end of the treatment period.

When vehicle controls became diabetic the experiment was ended and organs were collected for further analysis. When pancreata were used for FACS analysis, mice were perfused with 0.9% NaCl and 2 mg/mL Heparin after ketamine/xylazine overdose. In a follow-up experiment, the treatment of the mice started either the day before the transfer or on the same day and the mice were killed maximum two days after having developed T1D.

### 4.5 RIP-LCMV-GP mouse model

LCMV Armstrong clone 53b was produced as described previously [62]. Mice were infected with a concentration of 10^4^ plaque-forming units LCMV. IMU-838 was administrated at 150 mg/kg in 100 μL PEG-400 or in 200 μL 8% Phosal/H_2_O via oral gavage starting one day before infection with LCMV. BG was measured in weekly intervals using a CodeFree glucometer from SD Biosensor Inc. Animals with BG concentrations higher than 300 mg/dL were considered diabetic. Mice were analyzed at d14 or d28 for the short-term experiment. In a follow-up experiment, all the mice were treated daily with 150 mg/kg IMU-838 or vehicle control for 14 days. The IMU-838 treated mice were then split into two subgroups: one group (IMU-short) did not receive further treatment and another one (IMU-long) received IMU-838 three times a week until the end of the study. Mice were divided based on their BG to get a similar BG range in all groups and organs were analyzed by FACS or Immunohistochemistry.

### 4.6 Isolation of T cells from murine lymphoid organs and pancreas

Single cell suspensions of lymph nodes (LN) and spleen were obtained by grinding through a 70 μm cell strainer in HBSS+ (HBSS supplemented with 5% FCS (Biowest) and 10 mM HEPES (VWR)). For splenocytes an erythrocytes lysis step was performed by adding 2 mL of 0.83% NH_4_Cl/H2O for 2 min or by adding 1 mL of 1xACK lysis buffer (1.5 M NH_4_Cl, 100 mM KHCO_3_, 10 mM EDTA-N2) for 3–5 min on ice.

Pancreas was homogenized at 2,500 rpm for 30 s using 1.4 mm Precellys ceramic beads (Bertin). The suspension and remaining tissue were passed and grinded through a 40 μm cell strainer. Cells were centrifuged at 300×*g* for 10 min to remove debris. After density gradient centrifugation with 30% vs. 70% Percoll (Cytvia) for 30 min at 500×*g* (acceleration 1, deceleration 1, room temperature) the interphase was collected in HBSS+.

### 4.7 Isolation of T cells from human peripheral blood

PBMCs were isolated from venous blood, buffy coats, or cone blood by density gradient centrifugation over lymphocyte separation medium (Ficoll Paque PLUS (GE Healthcare), Histopaque®-1077 Hybri-max (Sigma–Aldrich) or Lymphoprep™ (Stem Cell Technologies)). PBMCs were either resuspended and frozen in freezing medium (CryoStor CS10) or processed further. Human CD4^+^ T cells for Treg induction *in vitro* were isolated from fresh PBMCs via MACS enrichment with CD4 microbeads following the manufacturer's instructions.

### 4.8 Cell staining and flow cytometry for murine and human samples

Isolated cells were incubated with Fc-Block (BioLegend) for 5–10 min on ice and stained for surface markers for 20–30 min on ice. All monoclonal antibodies used for FACS stainings are listed in Table S2 (murine) and Table S3 (human). Dead cells were excluded by viability stains using Sytox Blue or Red Live Dead Stain (Thermo Fisher Scientific) for unfixed cells or fixable viability dye eFluor450 (Thermo Fisher Scientific) for fixed cells. To detect intracellular proteins cells were fixed using the Foxp3 Staining Buffer Set (Thermo Fisher Scientific) for 30 min to 1 h on ice followed by intracellular staining using intracellular antibodies for 30 min to 1 h. Before acquisition on the flow cytometer, cells were passed through a 40 μm strainer (NeoLab).

For extensive murine sorts, T cells were pre-enriched using CD4-Biotin antibodies, Streptavidin beads and MACS (magnetic activated cell sorting) following manufacturer's protocol (Miltenyi) on LS columns.

For cytokine staining, cells were stimulated in presence of 0.5 μg/mL PMA (abcam) and 0.5 μg/mL Ionomycin (Cayman Chemicals) in RMPI+ (RPMI (Life Technologies) supplemented with 10% FCS (Biowest), 1 mM sodium pyruvate (Sigma Aldrich), 50 mM β-mercaptoethanol (BioConcept), 1X non-essential amino acids (Biochrom AG), 100 U/mL penicillin and 100 μg/mL streptomycin (Sigma Aldrich) with 1.5 mM CaCl_2_ for 2–4 h. The protein transport inhibitor brefeldin A (Golgi Plug, BD) was added (1:1000) after 1–2 h.

Single cell suspensions from LNs and spleens of RIP-LCMV-GP mice were stimulated overnight with the immunodominant LCMV peptides GP33 (10 μg/mL, GenScript) for CD8 T cells and GP61 (10 μg/mL, GenScript) for CD4 cells in the presence of Brefeldin A (1 μg/mL, Sigma). Cells were stained for surface antigens. After fixation and permeabilization with PFA/saponin solution, cells were stained for intracellular targets.

For the sort for and the analysis of human T cell proliferation assays, cells were stained as described above. Dead cells were excluded by viability stain using 7-AAD (Actinomycin D, 7-Amino, BD Bioscience or Calbiochem). Cells were filtered through a 35 μm cell strainer prior to sorting. A semi-automated electronic 96 channel pipette (VIAFLO) was programmed to perform the staining steps directly in the 96 well plate.

Cells were acquired on BD FACS Aria II and III, BD LSR Fortessa, BD LSR II, BD FACSCanto, FACS Aria Fusion II (Beckton Dickinson) using FACSDiva software with optimal compensation and gain settings determined based on unstained and single-color stained samples or on MACSQuant flow cytometer (Miltenyi Biotec). Doublets were excluded based on SSC and FSC plots. Live cell populations were gated based on cell side and forward scatter and the exclusion of cells positive for viability stains. Cells were sorted for purity with the FACS AriaIII or FACS Aria Fusion II.

Further analysis was performed using FlowJo (v9-10, Becton Dickinson) or FlowLogic (v7.3, Inivai Technologies Pty Ltd.).

### 4.9 Human T cell proliferation assays

Thawed PBMCs (Influenza stimulation) or CD4^+^ FACS depleted PBMCs together with FACS sorted CD4^+^ T cells, naïve (CD45RA^+^CD45RO^−^) or memory (CD45RA^−^CD45RO^+^) (SEB stimulation) were stained with the proliferation dyes eF670 (eBioscience/Invitrogen) or CellTrace Violet (Thermo Fisher Scientific) by incubating at 37 °C for 10–20 min (representative sorting strategy in Fig. S1B). After incubating with RPMI medium (Thermo Fisher Scientific) + 5% human serum (Corning and Sigma–Aldrich) for up to 10 min and washing, cells were resuspended in RPMI Glutamax (Thermo Fisher Scientific) medium with 5% human serum to a concentration of 2 × 10^5^ cells (Influenza stimulation) or 1 × 10^5^ cells (SEB stimulation) per well. Cells were seeded in round bottom 96-well plates (Costar, Corning) and Influenza Vaccine (Begripal, 2014/2015 Influenza Impfstoff, final concentration of 1 μL/mL) or SEB (Sigma–Aldrich, final concentration of 10 ng/mL) were added. IMU-935 and IMU-838 were added to the concentrations 5 nM or 5–10 μM, respectively.

### 4.10 Murine Treg induction assay *in vitro*

For murine Treg induction assays *in vitro*, 10,000 murine live naïve CD4^+^CD25^−^CD44^low^ T cells or activated CD4^+^CD25^−^CD44^high^GFP^−^ T cells were sort purified as described before. Activated T cells were sorted from Foxp3^GFP^ Balb/c mice by pregating on CD25^−^GFP^-^ T cells to avoid contaminating Tregs (representative sorting strategy in Fig. S3, A and C). Naïve T cells from RORγt^GFP^Foxp3^RFP^ mice were sorted as GFP^−^RFP^-^. Cells were resuspended in RPMI+ and 100 U/mL recombinant human IL-2 (Peprotech) and seeded in a 96-well plate pre-coated with 5 μg/mL anti-CD3 and 5 μg/mL anti-CD28 in 0.1 M sodium bicarbonate coating buffer, pH 8.5. Three different culturing conditions were used. First, for subimmunogenic TCR stimulation cells were transferred into uncoated plates after 18 h and cultured for an additional 36 h. Secondly, cells were continuously cultured for 54 h (continuous TCR stimulation). Thirdly, for Treg induction in presence of pro-inflammatory cytokines, cells were cultured using subimmunogenic TCR stimulation in presence of 10 ng/mL recombinant human IL-6, IFN-y and IL-1β (all PeproTech). Unless otherwise stated subimmunogenic Treg induction was used. IMU-935, IMU-838 and cedirogant were provided by Immunic Therapeutics. IMU-935, IMU-838, Tef (Sigma–Aldrich) and cedirogant (ABBV-157) were dissolved in DMSO and added at the start of the cell culture. Unless otherwise indicated following concentrations were used: IMU-935 1 μM, IMU-838 50 μM, Tef 7.5 μM, cedirogant 10 μM. For vehicle controls, DMSO with the respective concentrations were used. For experiments with uridine (Sigma Aldrich), uridine was dissolved in water and further diluted with RPMI + to the indicated concentrations.

### 4.11 Human Treg induction assay *in vitro*

For Treg inductions naïve CD4^+^ T cells (CD4^+^CD45RA^+^CD45RO^−^CD25^−^CD127^+^ T cells) or previously activated T cells (CD4^+^CD45RA^−^CD45RO^+^CD25^−^CD127^+^ T cells) were sorted for purity and 50,000 cells/well cultured in X-Vivo Medium (Lonza) supplemented with 2 mM glutamine, 50 U/mL penicillin, 50 mg/mL streptomycin (Sigma Aldrich) and 5% (v/v) heat-inactivated human AB serum (Invitrogen) (X-Vivo+) and recombinant human IL-2 (100 U/mL, Peprotech) similar to the murine Treg induction assay (representative sorting strategy in Fig. S2A). 96 well U-bottom plates were coated with 5 μg/mL anti-CD3 and 15 μg/mL anti-CD28 (subimmunogenic TCR stimulation) or 0.01 μg/mL anti-CD3 and 5 μg/mL anti-CD28 (continuous TCR stimulation). 10 ng/mL recombinant human IL-6 (Peprotech) was supplemented to subimmunogenic Treg induction assays when culturing in presence of the pro-inflammatory cytokine IL-6. IMU-935 and IMU-838 and respective vehicle controls were added 12 h after the start of the culture at concentration of 3.5 nM or 201 nM, respectively. To check for the induced Treg functionality, IMU-935 and respective control were added at a concentration of 1 nM and after continuous TCR stimulation, the cells were stimulated with in presence of 0.5 μg/mL PMA (abcam) and 0.5 μg/mL Ionomycin (Cayman Chemicals) in X-Vivo + medium for 4 h in the incubator. The protein transport inhibitor brefeldin A (Golgi Plug, BD) was added (1:1000) after 2 h. Cells were analyzed via flow cytometry.

### 4.12 Immunofluorescence analysis

Dissected pancreata were embedded in Tissue Tek O.C.T. Compound and frozen on dry ice. Tissues were cut using a Leica CM3050 S Cryostat to 7–8 μm thick sections. Tissue sections were thawed and fixed using 10% NBF or Ethanol/Acetone 1:1 followed by a permeabilization using 0.2% (v/v) Triton-X and blocking with 10% (v/v) FCS. The primary antibodies, Armenian hamster anti-CD3 (1:100, Invitrogen) and rabbit anti-glucagon (1:1200, abcam) or only rabbit anti CD3(1:300, DAKO) in the flex polyclonal anti-insulin ready to use solution (DAKO) were incubated overnight at 4 °C. The secondary antibodies, goat anti-guinea pig AF488, goat anti-rabbit antibody AF647 (both 1:500, Dianova) wih or without goat anti-Armenian hamster AF555 (1:500, Invitrogen) were incubated for 1 h at room temperature. Nuclei were counterstained with Hoechst 33,342 (Invitrogen). Negative control slides were incubated with secondary antibodies. Sections were imaged using the Phenoimager Fusion (Akoya Biosciences) with 20x for quantification and representative images were taken with a confocal microscope with 20x objective (Zeiss, LSM880). A blinded analysis was performed using Qupath and a pixel classifier was trained to identify islets with residual insulin production (v0.4.4). The mean intensity of CD3 or insulin for the selected region/islets or the area was used for analysis.

### 4.13 Immunohistochemistry staining

Pancreatic tissue sections were fixed in ethanol or ethanol/acetone (1:1) at −20 °C. The sections were blocked with 10% (v/v) FCS in PBS. Primary antibodies used were rat anti-CD8b (1:100, BioLegend), rabbit anti-insulin (1:4,000, Abcam). As secondary antibodies, biotinylated anti-rat (1:500, Vector) and biotinylated anti-rabbit (1:500, Vector) were used. After ABC complex (Vector Laboratories), DAB substrate was used to develop colour and nuclei were counterstained with hematoxylin. Images of pancreas sections were acquired with a BZ-X810 microscope (Keyence) with a 20× objective. The software BZII Analyzer (Keyence) was used for the quantification of the immunohistochemistry images.

### 4.14 Insulitis scoring

Insulitis was scored according to the following system: Score 0: no or very minor insulitis, only very few infiltrating cells; score 1: mild to moderate insulitis, 25–50% infiltrates large parts with intact beta cells; score 2: considerable insulitis, 50–75% infiltrates, still some parts with intact beta cells; score 3: massive insulitis, 75–100% infiltrates, only few remaining beta cells producing insulin, islet scar.

### 4.15 Quantitative real time PCR

RNA was extracted with RNAdvance Cell v2 Kit (Beckman Coulter). For that, up to 200,000 cells were sorted as live CD4^+^, CD25^+^CD4^+^ (Tregs), CD44^low^CD25^−^CD4^+^ (naïve) or CD44^high^CD25^−^CD4^+^ (activated). cDNA was reverse transcribed using iScript Advanced cDNA Synthesis Kit (BioRad), followed by RT-qPCR using the CFX96 Touch Real-Time PCR Detection System and SsoFast™ Evagreen Supermix reagents (BioRad). The data were processed with Bio-Rad CFX Manager 3.1 Software (BioRad) and obtained Cq values were normalized to the expression of *Histone*. For primer sequences, see Table S1.

### 4.16 Statistics

All data were analyzed using GraphPad Prism (v8-10) and represented as box-and-whiskers plots or bar graphs with mean and standard deviation (SD). Each data point represents one biological replicate (one mouse or human donor). For *in vitro* studies, experiments were performed in two or three technical replicates, with exception of the isotype control. For normally distributed data, unpaired Student's t-test or a Student's t-test for paired values was used to compare means of two independent groups or comparing values from the same sample which was treated under different conditions, respectively. For multiple comparison, one-way ANOVA with Tukey's multiple comparison test or two-way ANOVA with Sidak's multiple comparisons test was used. Diabetes incidences were plotted in a survival curve and significance was tested using a Mantel–Cox test. For all tests, a two-tailed P value < 0.05 was considered as significant. Statistical significance is shown as ∗ = P < 0.05; ∗∗ = P < 0.01; ∗∗∗ = P < 0.001, ∗∗∗∗ = P < 0.0001.

## CRediT authorship contribution statement

**Hannah Hipp:** Conceptualization, Data curation, Formal analysis, Investigation, Methodology, Supervision, Visualization, Writing – original draft. **Camilla Tondello:** Data curation, Formal analysis, Investigation, Methodology, Validation, Visualization, Writing – review & editing. **Hanna Gmehling:** Formal analysis, Investigation. **Lena K. Scholz:** Formal analysis, Investigation. **Antigoni Stavridou:** Formal analysis, Investigation, Methodology. **Maike Becker:** Visualization, Writing – review & editing, Conceptualization, Investigation, Project administration, Supervision. **Anne-Marie Bührer:** Investigation. **Edith Hintermann:** Investigation. **Sandra M. Dirschl:** Investigation. **Till M. Johannsmann:** Investigation. **Martin G. Scherm:** Visualization, Writing – original draft, Writing – review & editing. **Hella Kohlhof:** Writing – review & editing. **Isabelle Serr:** Writing – review & editing. **Urs Christen:** Formal analysis, Investigation, Methodology, Supervision, Writing – review & editing. **Carolin Daniel:** Conceptualization, Funding acquisition, Methodology, Resources, Supervision, Writing – original draft, Writing – review & editing.

## Funding

CD holds a professorship grant from the Excellence Program for Outstanding Female Scientists from the Helmholtz Association and is supported by a Research Group at Helmholtz Zentrum München, through a membership in the CRC1054 of the Deutsche Forschungsgemeinschaft Project-ID 210592381–SFB 1054 (B11), through a membership in the TRR355 of the Deutsche Forschungsgemeinschaft Project-ID 490846870-TRR355/1 (A02), and the Breakthrough T1D (formerly JDRF) Project-ID 3-SRA-2023-1420-S-B. CD, MB and TMJ receive funding by the Helmholtz Munich Innovation and Translation Fund 2023 and 2025. TMJ was funded by the 10.13039/501100001656Helmholtz Association - Initiative and Networking Fund (IVF) as part of the International Helmholtz Research School for Diabetes. SMD and TMJ are supported by a membership of iTarget2.0 of the Ludwig-Maximilians-Universität München. IS is supported through a membership in the TRR355 of the Deutsche Forschungsgemeinschaft Project-ID 490846870-TRR355/1 (B02). AS was supported by the Transcampus IRTG 2251: “Immunological and Cellular Strategies in Metabolic Disease” (ICSMD) (Deutsche Forschungsgemeinschaft, Project-ID: 288034826). The authors are supported by a membership of the German Center for Diabetes Research (DZD) with grants to CD, MB and IS. This research was supported by funding of the Fritz Bender Stiftung granted to CD. Open Access funding enabled and organized by Projekt DEAL.

## Declaration of competing interest

The authors declare the following financial interests/personal relationships which may be considered as potential competing interests: Carolin Daniel reports equipment, drugs, or supplies was provided by Immunic AG. Hella Kohlhof is an employee of Immunic AG, own shares and stock-options of the parent company of Immunic AG, Immunic Inc and is the holder of patents. All other authors declare they have no competing interests. If there are other authors, they declare that they have no known competing financial interests or personal relationships that could have appeared to influence the work reported in this paper.

## Data Availability

Data will be made available on request.

## Glossary

**Type 1 Diabetes (T1D)**: is a chronic condition in which the pancreas produces little or no insulin due to the autoimmune destruction of insulin-producing beta cells
**Regulatory T Cells (Tregs)**: are a subset of T cells that modulate the immune system, maintain tolerance to self-antigens, and prevent autoimmune disease by suppressing the immune response
**Forkhead Box P3 (Foxp3)**: is a protein crucial for the normal function of regulatory T cells; it acts as a transcription factor that controls the expression of genes involved in immune regulation
**Insulin Autoantibodies (IAA)**: are autoantibodies targeting insulin, often present in individuals with type 1 diabetes; their presence can indicate an autoimmune response against pancreatic beta cells
**Dihydroorotate Dehydrogenase (DHODH)**: is an enzyme involved in the *de novo* synthesis of pyrimidines, which are essential for DNA and RNA synthesis; it is a target for drugs that aim to modulate the immune system in autoimmune diseases and cancer
