# Supplementary material for: Inhibition of pyrimidine de novo synthesis fosters Treg cells and reduces diabetes development in models of Type 1 Diabetes
Source: Mol Metab. 2025 Jul 22;100:102218. doi: 10.1016/j.molmet.2025.102218 (PMC12791205; doi:10.1016/j.molmet.2025.102218)
Supplement: Multimedia component 1 [file mmc1.pdf]

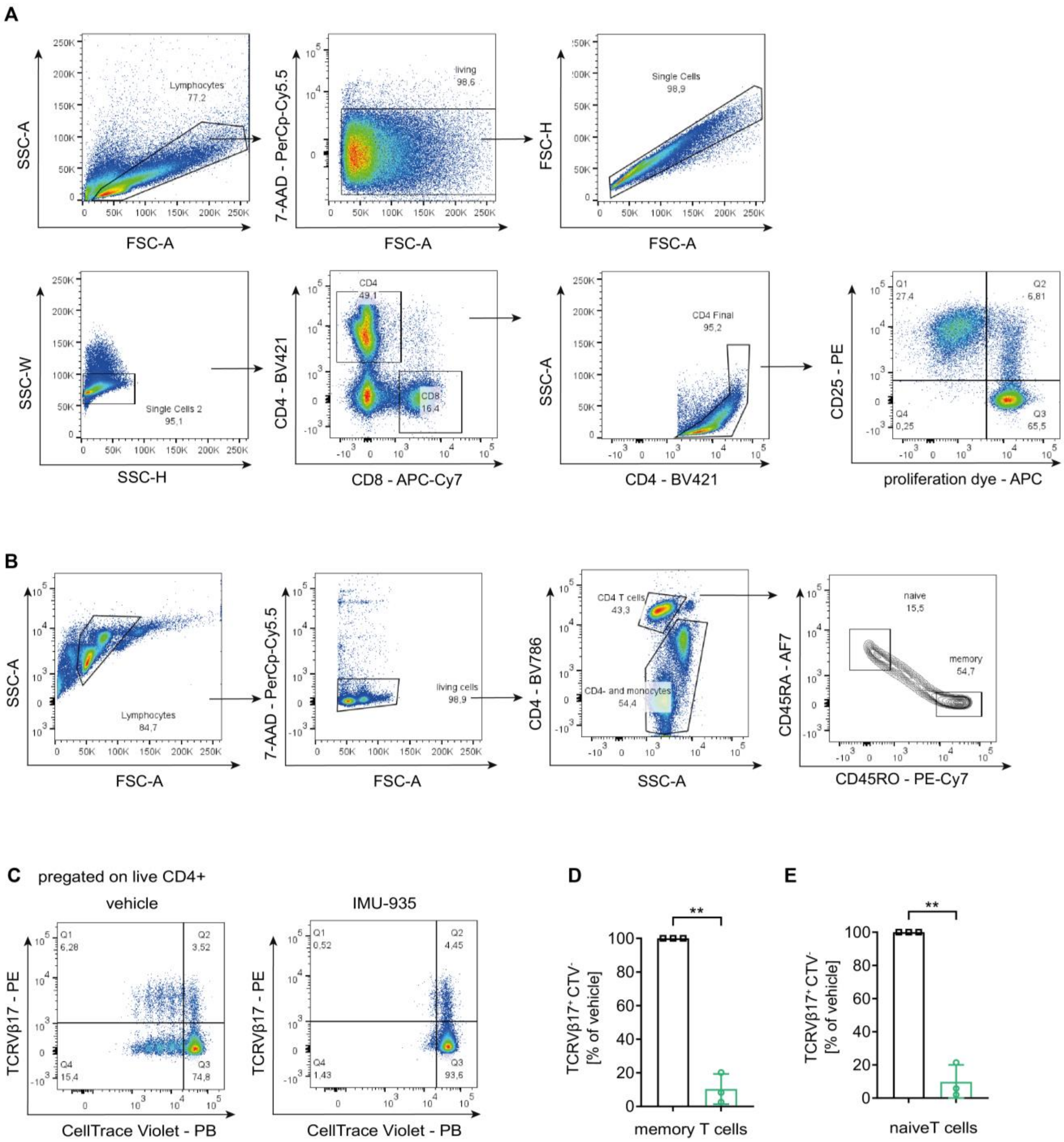
A
SSC-A
FSC-A
Lymphocytes
77,2
7-AAD - PerCp-Cy5.5
living
98,6
FSC-H
Single Cells
98,9
SSC-W
SSC-H
Single Cells 2
95,1
CD4 - BV421
CD8 - APC-Cy7
CD4
49,1
CD8
16,4
SSC-A
CD4 Final
95,2
CD25 - PE
proliferation dye - APC
Q1
27,4
Q2
6,81
Q3
65,5
Q4
0,25
B
SSC-A
FSC-A
Lymphocytes
84,7
7-AAD - PerCp-Cy5.5
living cells
98,9
CD4 - BV786
SSC-A
CD4 T cells
43,3
CD4- and monocytes
54,4
CD45RA - AF7
CD45RO - PE-Cy7
naive
15,5
memory
54,7
C
pregated on live CD4+
vehicle
IMU-935
TCRVβ17 - PE
CellTrace Violet - PB
Q1
6,28
Q2
3,52
Q3
74,8
Q4
15,4
Q1
0,52
Q2
4,45
Q3
93,6
Q4
1,43
D
TCRVβ17⁺CTV⁻
[% of vehicle]
memory T cells
E
TCRVβ17⁺CTV⁻
[% of vehicle]
naiveT cells
**

Fig. S1. IMU-935 reduces responses of naïve and memory $CD4^+$ T cells towards SEB antigens.

(**A**) Representative gating strategy to analyze the proliferation of human $CD4^+$ T cells in response to influenza vaccine in T cell proliferation assays.

(**B**) Representative gating strategy to sort naïve ($CD45RA^-CD45RO^+$), memory ($CD45RA^+CD45RO^-$) $CD4^+$ T cells and $CD4^+$ FACS depleted PBMC.

(**C-E**) Human T cell proliferation assay to assess TCRVβ-specific $CD4^+$ T cell response to SEB. (**C**) Representative FACS plots. Analysis of proliferating TCRVβ17$^+$CellTrace Violet CTV$^+$ $CD4^+$ T cells in response to SEB of (**D**) memory or (**E**) naïve $CD4^+$ T cells with 5nM of IMU-935 or a vehicle control, n = 3.

Each data point represents one subject. Student's t test. **=p<0.01.

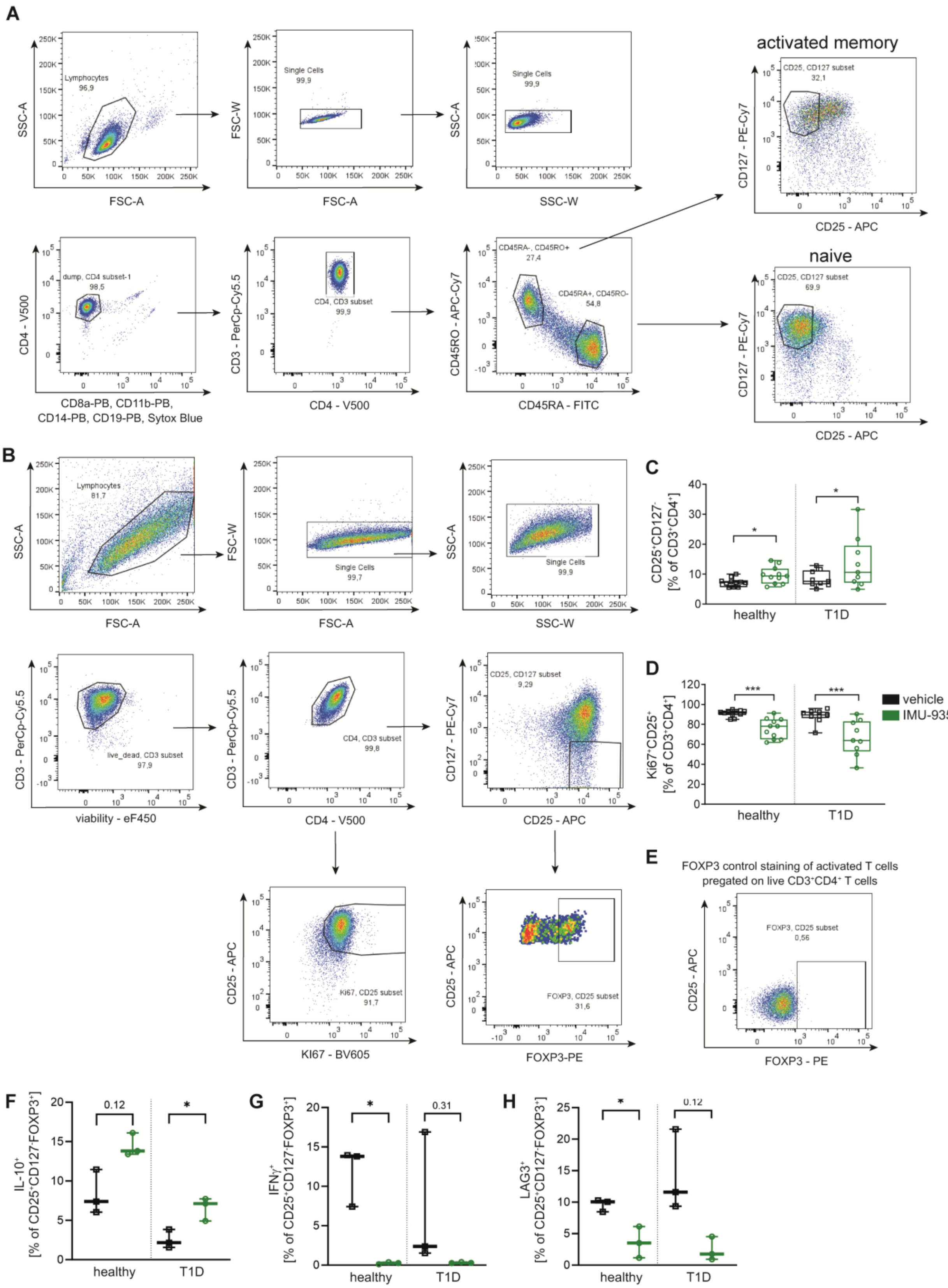
A
B
C
D
E
F
G
H
activated memory
naive
SSC-A
FSC-A
FSC-W
SSC-W
Lymphocytes
Single Cells
CD4 - V500
CD8a-PB, CD11b-PB, CD14-PB, CD19-PB, Sytox Blue
CD3 - PerCp-Cy5.5
CD45RO - APC-Cy7
CD45RA - FITC
CD127 - PE-Cy7
CD25 - APC
viability - eF450
KI67 - BV605
FOXP3-PE
healthy
T1D
vehicle
IMU-935
FOXP3 control staining of activated T cells pregated on live CD3⁺CD4⁺ T cells
FOXP3 - PE
0.12
0.31

Fig. S2. IMU-935 enhances $CD25^+CD127^-$ Tregs in human Treg induction *in vitro*.

(**A**) Representative gating strategy for human naïve and previously activated memory T cells.

(**B**) Representative FACS plots for the analysis of human Treg induction *in vitro*.

(**C, D**) Human Treg induction *in vitro* using subimmunogenic TCR stimulation and naïve T cells isolated from PBMCs of healthy subjects or T1D donors in presence or absence of 3.5 nM IMU-935. Summary plots for the frequency of (**C**) induced $CD25^+CD127^-CD3^+CD4^+$ Tregs and (**D**) proliferating $CD25^+Ki67^+CD3^+CD4^+$ T cells. Healthy n = 10, T1D n = 9.

(**E**) Representative FACS plots of FOXP3 staining among sorted activated human T cells, pregated on $CD3^+CD4^+$ T cells.

(**F-H**) Human Treg induction *in vitro* using continuous TCR stimulation and naïve T cells isolated from PBMCs of healthy subjects or T1D donors in presence or absence of 1 µM IMU-935. Summary plots for the frequency of (**F**) $IL\text{-}10^+$ producing cells, (**G**) $IFN\gamma^+$ producing cells and (**H**) $LAG3^+$ expressing cells of induced $CD25^+CD127^-FOXP3^+$ Tregs. Healthy n = 3, T1D n = 3. Each data point represents one subject, experiments were performed in 2-3 technical replicates. Student's *t* test. *=$p<0.05$; ***=$p<0.001$.

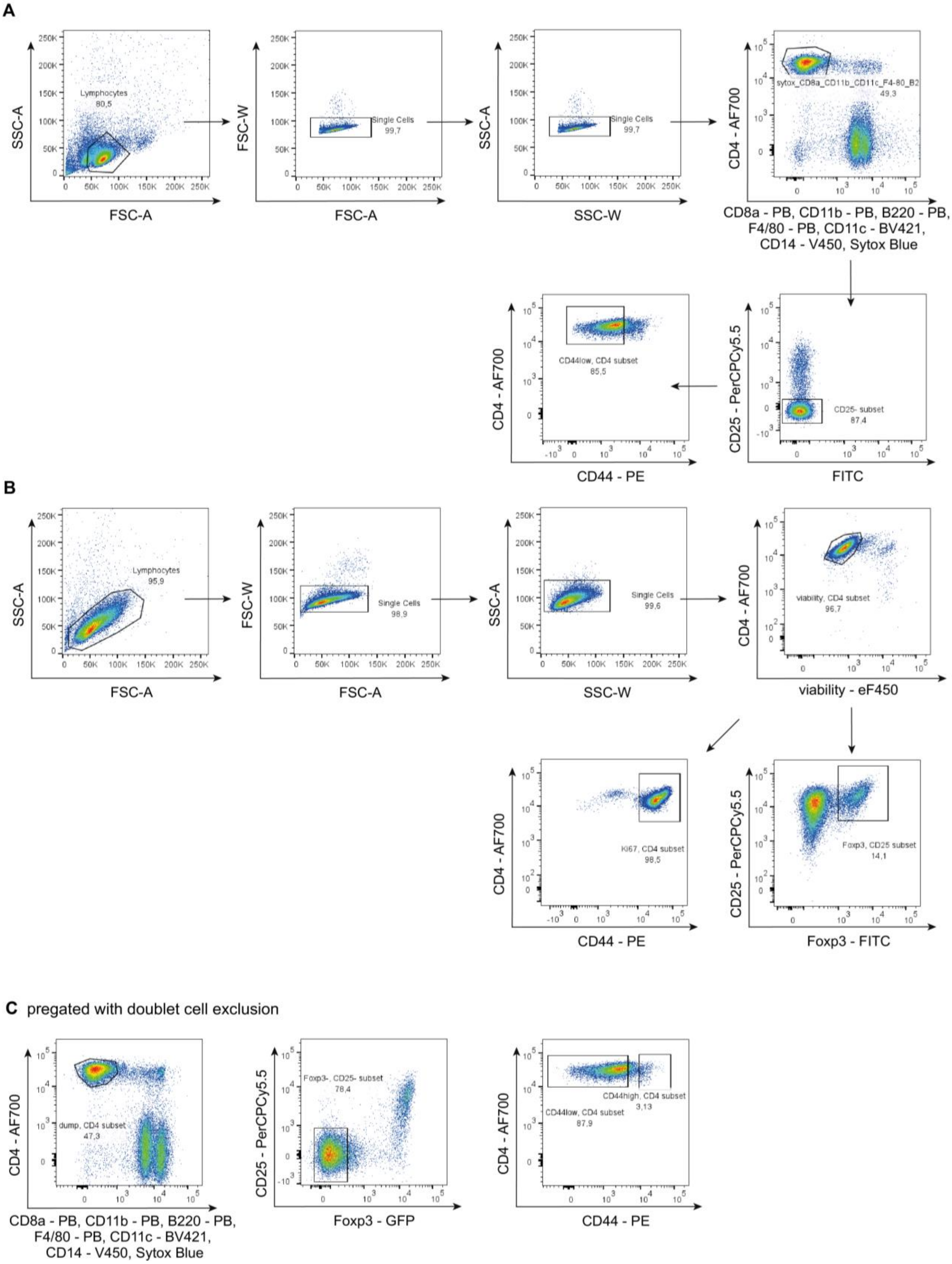
A
Lymphocytes
80,5
SSC-A
FSC-A
FSC-W
Single Cells
99,7
SSC-A
SSC-W
Single Cells
99,7
CD4 - AF700
sytox_CD8a_CD11b_CD11c_F4-80_B2
49,3
CD8a - PB, CD11b - PB, B220 - PB, F4/80 - PB, CD11c - BV421, CD14 - V450, Sytox Blue
CD4 - AF700
CD44low, CD4 subset
85,5
CD44 - PE
CD25 - PerCPCy5.5
CD25- subset
87,4
FITC
B
Lymphocytes
95,9
SSC-A
FSC-A
FSC-W
Single Cells
98,9
SSC-A
SSC-W
Single Cells
99,6
CD4 - AF700
viability, CD4 subset
96,7
viability - eF450
CD4 - AF700
Ki67, CD4 subset
98,5
CD44 - PE
CD25 - PerCPCy5.5
Foxp3, CD25 subset
14,1
Foxp3 - FITC
C pregated with doublet cell exclusion
CD4 - AF700
dump, CD4 subset
47,3
CD8a - PB, CD11b - PB, B220 - PB, F4/80 - PB, CD11c - BV421, CD14 - V450, Sytox Blue
CD25 - PerCPCy5.5
Foxp3-, CD25- subset
78,4
Foxp3 - GFP
CD4 - AF700
CD44high, CD4 subset
3,13
CD44low, CD4 subset
87,9
CD44 - PE

Fig. S3. Gating strategy for FACS sorting and analysis of murine T cells.

(**A**) Representative FACS plots for the sort of murine naïve T cells.

(**B**) Representative FACS plots for the analysis of $CD25^{+}Foxp3^{+}$ Tregs and $Ki67^{+}$ T cells after Treg induction *in vitro*.

(**C**) Representative FACS plots for the sort of naïve and activated T cells from $Foxp3^{GFP}$ Balb/c mice.

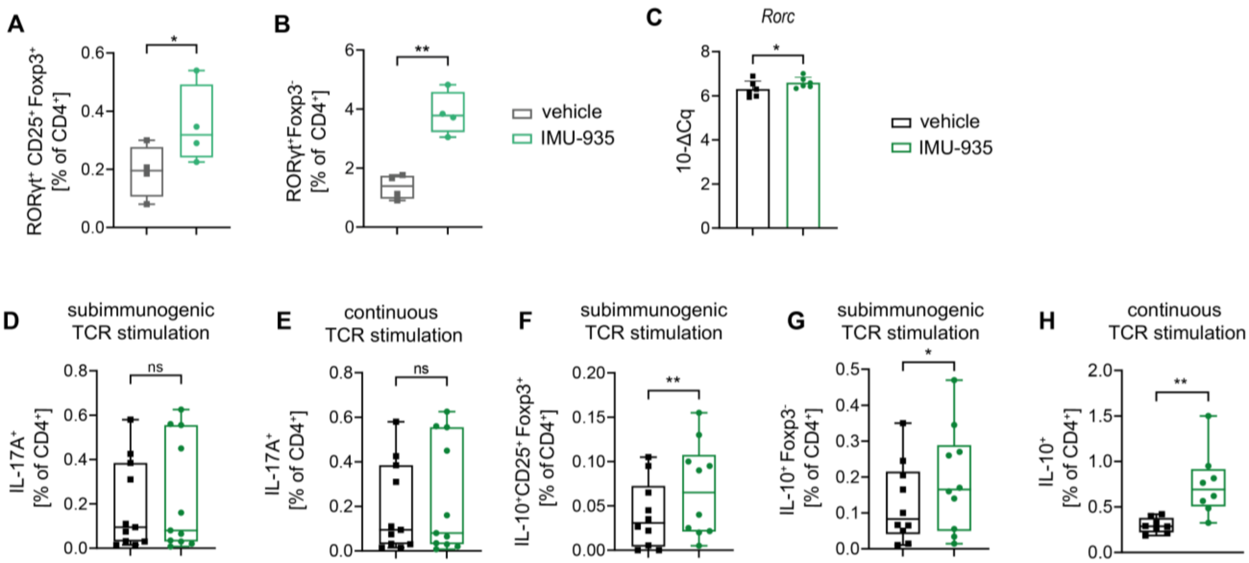

Fig. S4. IMU-935 enhances RORγt expression while fostering an anti-inflammatory phenotype after Treg induction *in vitro*.

(**A, B**) Murine Treg induction *in vitro* using subimmunogenic TCR stimulation and naïve T cells from LN of $ROR\gamma t^{GFP}Foxp3^{RFP}$ reporter mice in presence of 1 µM IMU-935 or the vehicle control. Summary plot for (**A**) $ROR\gamma t^{+}CD25^{+}Foxp3^{+}$ Tregs and (**B**) $ROR\gamma t^{+}Foxp3^{-}$ T cells, n = 4.

(**C**) qPCR analysis of *Rorc* gene expression of sorted live $CD4^{+}$ T cells after Treg induction *in vitro* using continuous TCR stimulation and naïve T cells isolated from LN of NOD mice in presence or absence of IMU-935. Gene expression was normalized to *Histone H3*, n = 6.

(**D, E**) Frequency of IL-17^{+} T cells after Treg induction *in vitro* using naïve T cells isolated from LN of NOD mice and (**D**) subimmunogenic (n = 11) or (**E**) continuous TCR stimulation (n = 7) in presence of 1 µM IMU-935 or the vehicle control.

(**F**) Frequency of $IL10^{+}CD25^{+}Foxp3^{+}$ Tregs and (**G**) $IL10^{+}Foxp3^{-}$ T cells after Treg induction *in vitro* using subimmunogenic TCR stimulation and naïve T cells isolated from LN of NOD mice in presence of 1µM IMU-935 or the vehicle control, n = 10.

(**H**) Frequency of $IL10^{+}$ cells among all live $CD4^{+}$ T cells after Treg induction *in vitro* using continuous TCR stimulation and naïve T cells isolated from LN of NOD mice in presence of 1 µM IMU-935 or the vehicle control, n = 8.

Each data point represents one subject, experiments were performed in 2-3 technical replicates. Student's t test. *=$p<0.05$; **=$p<0.01$; ***=$p<0.001$.

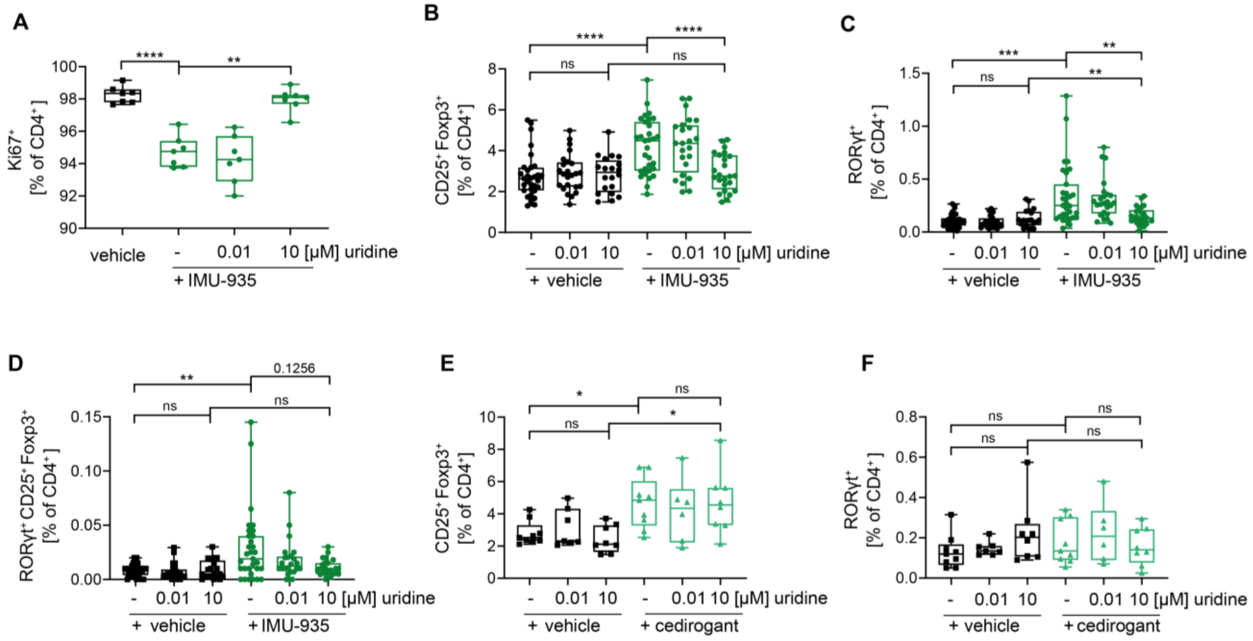

Fig. S5. Excess of uridine diminishes effects of IMU-935 on Treg induction *in vitro*.

(**A**) Frequency of proliferating Ki67$^+$ T cells after murine subimmunogenic Treg induction *in vitro* using naïve T cells isolated from LN of NOD mice in presence or absence of 1 μM IMU-935 and increasing concentrations of uridine, n = 7.

(**B-D**) *In vitro* Treg induction in the presence of the proinflammatory cytokines IL-6, IFN-γ and IL-1β and using naïve T cells isolated from LN of NOD mice with or without 1 μM IMU-935 and increasing concentrations of uridine. Summary plots for the frequency of (**B**) CD25$^+$Foxp3$^+$ Tregs (**C**) RORγt$^+$ T cells and (**D**) Rorγt$^+$CD25$^+$Foxp3$^+$ Tregs, n = 20-31.

(**E, F**) *In vitro* Treg induction in the presence of the proinflammatory cytokines IL-6, IFN-γ and IL-1β using naïve T cells isolated from LN of NOD mice with or without the RORγt inverse agonist cedirogant (10 μM) and increasing concentrations of uridine. Frequency of (**E**) CD25$^+$Foxp3$^+$ Tregs and (**F**) RORγt$^+$ T cells, n = 6-9.

Each data point represents one subject, experiments were performed in 2-3 technical replicates. One-way ANOVA with Tukey's post hoc test for multiple comparisons. *=p<0.05; **=p<0.01; ***=p<0.001; ****=p<0.0001.

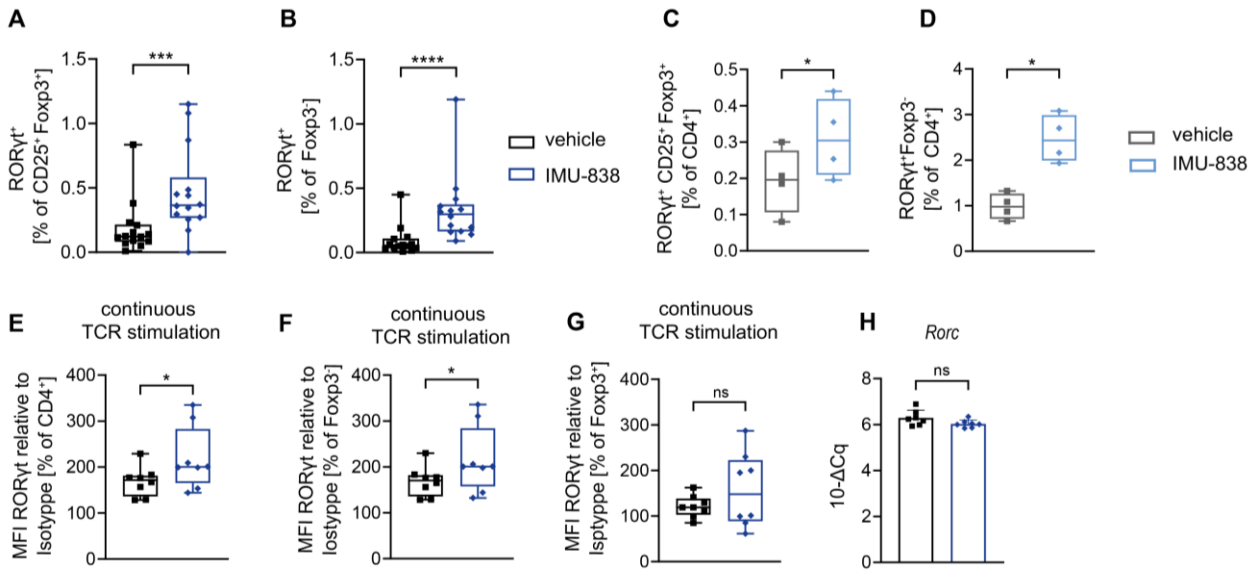

Fig. S6. IMU-838 enhances RORγt$^+$ Treg and non-Treg frequency after Treg induction *in vitro*.

(**A, B**) Frequency of RORγt in (**A**) CD25$^+$Foxp3$^+$ Tregs or (**B**) Foxp3$^-$ non-Tregs after Treg induction *in vitro* using subimmunogenic TCR stimulation and naïve T cells from LN of NOD mice in presence or absence of 50 µM IMU-838, n = 14.

(**C, D**) Frequency of (**C**) RORγt$^+$CD25$^+$Foxp3$^+$ Tregs or (**D**) RORγt$^+$Foxp3$^-$ non-Tregs after Treg induction *in vitro* using subimmunogenic TCR stimulation and naïve T cells from LN of RORγt^{GFP}Foxp3^{RFP} reporter mice in presence or absence of 50 µM IMU-838, n = 4.

(**E-G**) Isotype-normalized MFI of RORγt in (**E**) total CD4$^+$ T cells, (**F**) Foxp3$^-$ non-Tregs and (**G**) CD25$^+$Foxp3$^+$ Tregs after Treg induction *in vitro* using continuous TCR stimulation and naïve T cells from LN of NOD mice in presence or absence of 50 µM IMU-838, n = 8.

(**H**) qPCR analysis of *Rorc* gene expression of sorted live CD4$^+$ T cells after Treg induction *in vitro* using continuous TCR stimulation and naïve T cells isolated from LN of NOD mice in presence or absence of 50 µM IMU-838. Gene expression was normalized to *Histone H3*, n = 7.

Each data point represents one subject, experiments were performed in 2-3 technical replicates. Student's *t* test. *=p<0.05; ***=p<0.001; ****=p<0.0001.

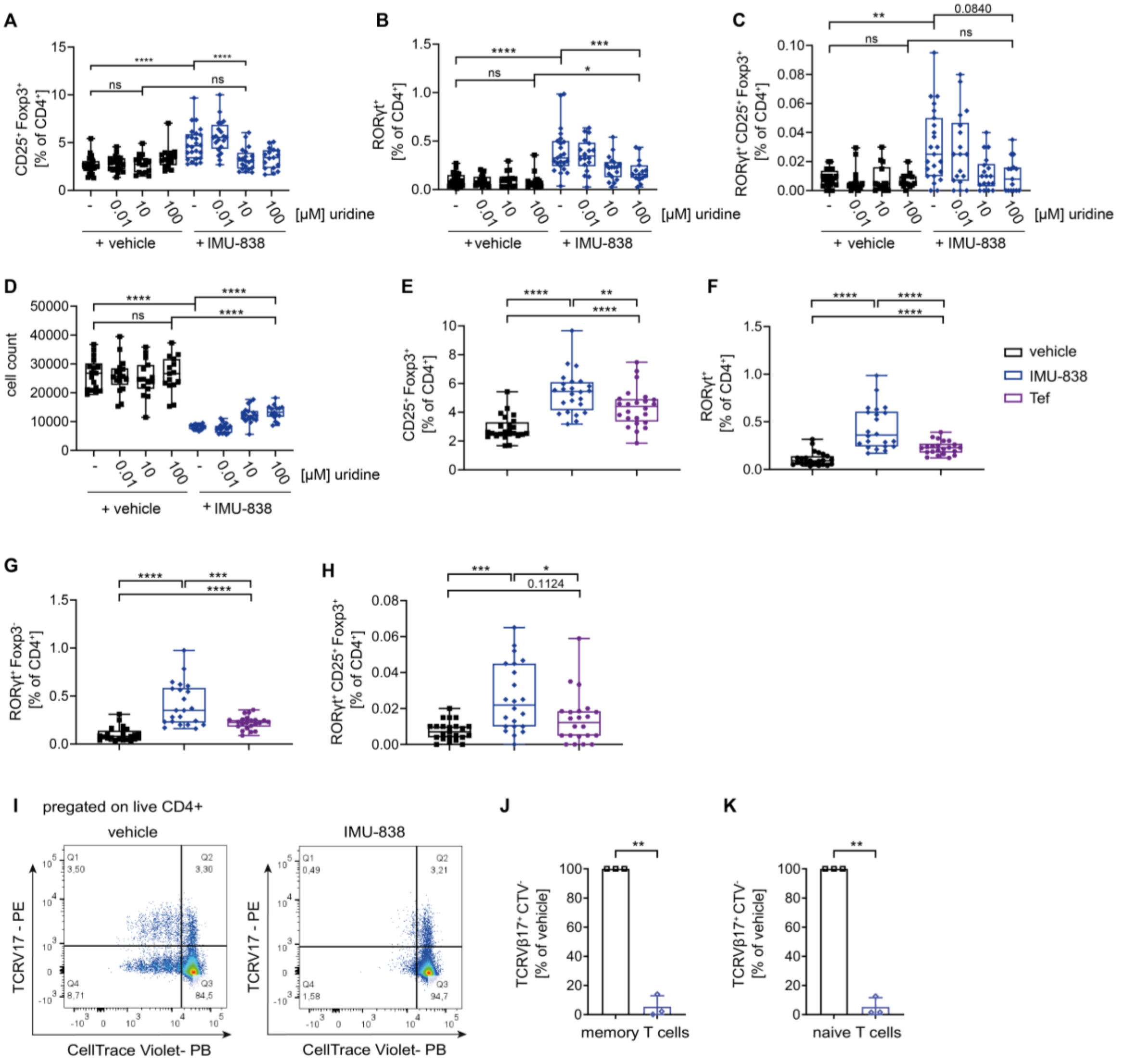

Fig. S7. DHODH inhibition enhances murine Treg induction *in vitro*.

(**A-D**) *In vitro* Treg induction in the presence of the proinflammatory cytokines IL-6, IFN-γ and IL-1β and using naïve T cells isolated from LN of NOD mice with or without 50 μM IMU-838 and increasing concentrations of uridine. Summary plots for the frequency of (**A**) CD25$^+$Foxp3$^+$ Tregs (**B**) RORγt$^+$ T cells (**C**) Rorγt$^+$CD25$^+$Foxp3$^+$ Tregs (**D**) cell count of total recovered cells, n = 14-26 experiments with 2-3 technical replicates.

(**E-H**) Frequency of (**E**) CD25$^+$Foxp3$^+$ Tregs, (**F**) RORγt$^+$ T cells, (**G**) RORγt$^+$Foxp3$^-$ non-Tregs and (**H**) RORγt$^+$CD25$^+$Foxp3$^+$ Tregs after Treg induction *in vitro* in the presence of the proinflammatory cytokines IL-6, IFN-γ and IL-1β and using naïve T cells isolated from

LN of NOD mice with or without 50 μM IMU-838 or 7.5 μM Teriflunomide (Tef), n = 22-24 experiments with 2-3 technical replicates.

(**I-K**) Human T cell proliferation assay to assess TCRVβ-specific $CD4^+$ T cell response to SEB. (**I**) Representative FACS plots. Analysis of proliferating TCRVβ17^+CellTrace Violet CTV^+ $CD4^+$ T cells in response to SEB of (**J**) memory or (**K**) naïve $CD4^+$ T cells with 10 μM of IMU-838 or a vehicle control, n = 3. Each data point represents one subject, (A-H) experiments were performed in 2-3 technical replicates. (A-H) One-way ANOVA with Tukey's post hoc test for multiple comparisons, (J, K) Student's t-test. *=p<0.05; **=p<0.01; ***=p<0.001; ****=p<0.0001.

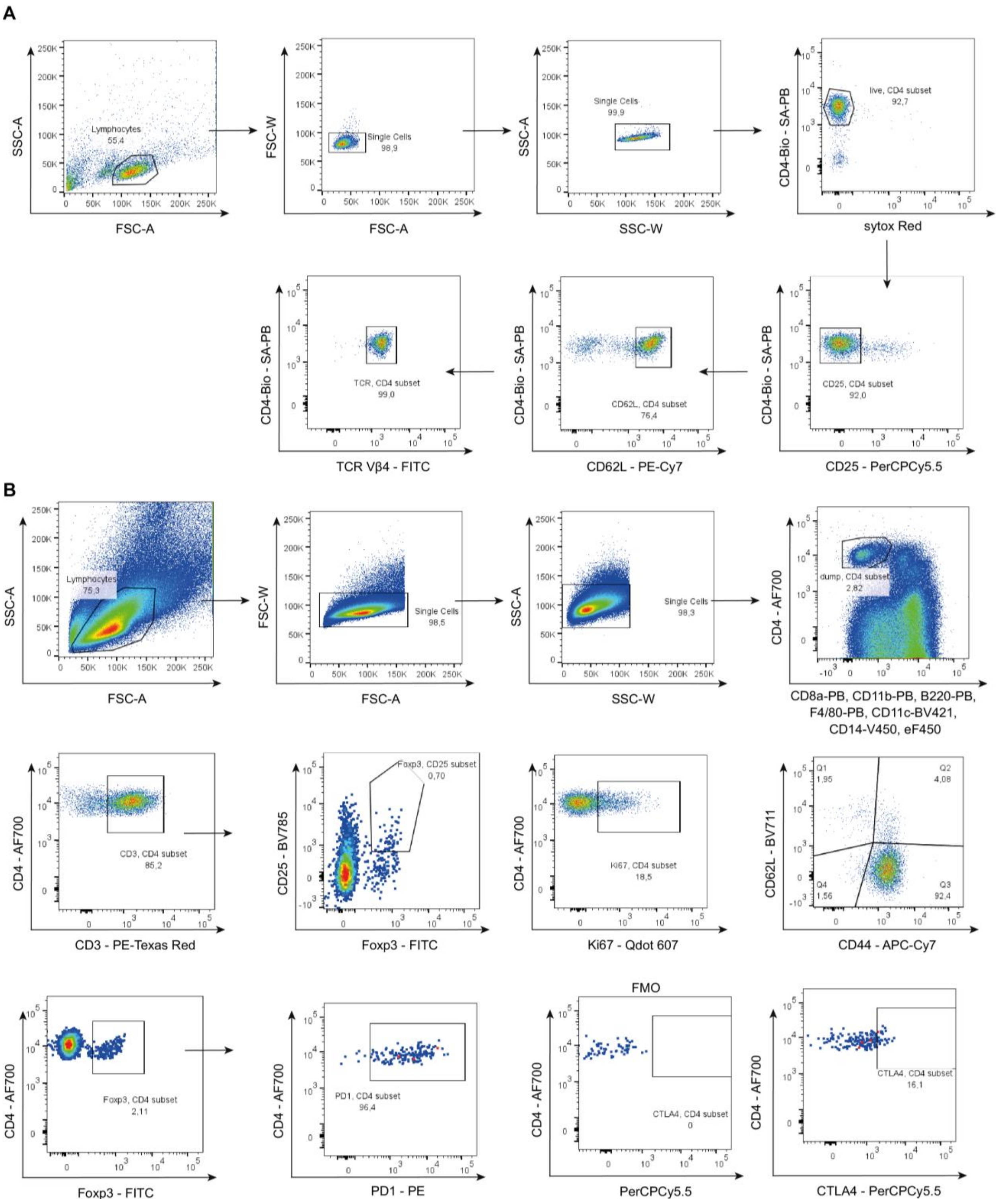
A
SSC-A
FSC-A
Lymphocytes
55,4
FSC-W
Single Cells
98,9
Single Cells
99,9
SSC-W
CD4-Bio - SA-PB
live, CD4 subset
92,7
sytox Red
TCR, CD4 subset
99,0
TCR Vβ4 - FITC
CD62L, CD4 subset
76,4
CD62L - PE-Cy7
CD25, CD4 subset
92,0
CD25 - PerCPCy5.5
B
Lymphocytes
75,3
Single Cells
98,5
Single Cells
98,3
CD4 - AF700
dump, CD4 subset
2,82
CD8a-PB, CD11b-PB, B220-PB, F4/80-PB, CD11c-BV421, CD14-V450, eF450
CD3, CD4 subset
85,2
CD3 - PE-Texas Red
CD25 - BV785
Foxp3, CD25 subset
0,70
Foxp3 - FITC
Ki67, CD4 subset
18,5
Ki67 - Qdot 607
CD62L - BV711
Q1
1,95
Q2
4,08
Q4
1,56
Q3
92,4
CD44 - APC-Cy7
Foxp3, CD4 subset
2,11
Foxp3 - FITC
PD1, CD4 subset
96,4
PD1 - PE
FMO
CTLA4, CD4 subset
0
PerCPCy5.5
CTLA4, CD4 subset
16,1
CTLA4 - PerCPCy5.5

Fig. S8. Analysis of IMU-838 treatment in a T1D mouse model induced by adoptive transfer.

(**A**) Representative gating strategy for the FACS sort of live $CD4^{+}CD25^{-}CD62L^{+}TCRV\beta 4^{+}$ T cells from NOD BDC2.5 mice.

(**B**) Representative FACS plots for the gating strategy for the analysis from Fig. 5.

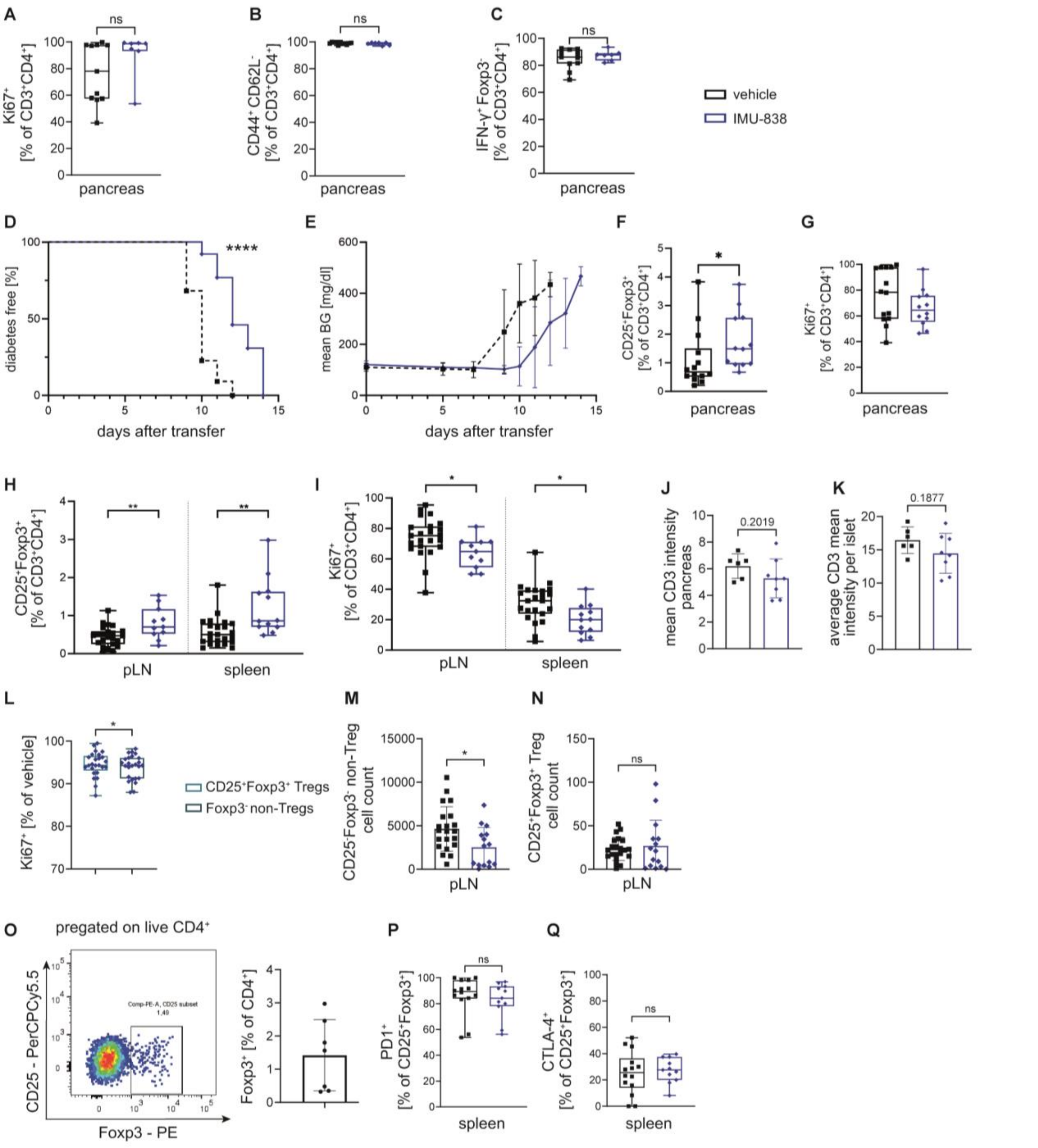

Fig. S9. Impact of IMU-838 treatment in a mouse model of accelerated T1D.

(**A-C**) Frequency of (**A**) proliferating Ki67$^+$CD3$^+$CD4$^+$ T cells, (**B**) activated CD44$^+$CD62L$^-$ CD3$^+$CD4$^+$ T cells and (**C**) IFNγ$^+$CD3$^+$CD4$^+$ T cells in the pancreas in mice treated with the vehicle control or IMU-838 in the mouse model of accelerated T1D, vehicle n = 11, IMU-838 n = 7.

(**D**) Follow up incidence study in the mouse model of accelerated T1D, vehicle n=22, IMU-838 n=13.

(**E**) Mean blood glucose values of the mice in D.

(**F, G**) Frequencies of (**F**) $CD25^{+}Foxp3^{+}$ T cells and (**G**) proliferating $Ki67^{+}CD3^{+}CD4^{+}$ T cells in the pancreas of mice treated with the vehicle control or IMU-838 in the mouse model of accelerated T1D, vehicle n = 14, IMU-838 n = 12.

(**H, I**) Frequencies of (**H**) $CD25^{+}Foxp3^{+}$ T cells and (**I**) of proliferating $Ki67^{+}CD3^{+}CD4^{+}$ T cells in the pLN and in the spleen of mice treated with the vehicle control or IMU-838 in the mouse model of accelerated T1D, vehicle n = 23, IMU-838 n = 11-12.

(**J**) Mean of the CD3 intensity in the whole pancreas quantified after immunofluorescence staining from pancreatic tissue slides from control mice or mice treated with IMU-838, vehicle n = 6, IMU-838 n = 8.

(**K**) Mean of the CD3 intensity averaged over all insulin-producing islets quantified after immunofluorescence staining from pancreatic tissue slides from control mice or mice treated with IMU-838, vehicle n = 6, IMU-838 n = 8.

(**L**) $Ki67^{+}CD25^{+}Foxp3^{+}$ Tregs or $Ki67^{+}Foxp3^{-}$ non-Tregs after *in vitro* Treg induction in the presence of the proinflammatory cytokines IL-6, IFN-γ and IL-1β and using naïve T cells isolated from LN of NOD mice with or without IMU-838, represented here as % of vehicle, n = 25.

Cell count of (**M**) $CD25^{-}Foxp3^{-}$ non-Tregs and (**N**) $CD25^{+}Foxp3^{+}$ Tregs in pLN of control or IMU-838-teated mice, vehicle n = 21, IMU-838 n = 15.

(**O**) Representative FACS plot and quantification for Foxp3 expression in sorted $CD4^{+}CD25^{-}CD62L^{+}TCRV\beta4^{+}$ T cells from NOD BDC2.5 mice, n = 7.

Frequency of (**P**) PD1 and (**Q**) CTLA-4 in Tregs isolated from spleen of control or IMU-838-treated mice, vehicle n = 14, IMU-838 n = 11.

Each data point represents one subject, (A-E, G-K) experiments were performed in up to seven independent experiments, (F) experiments were performed in 2-3 technical replicates. Student's *t* test. *=p<0.01; **=p<0.01.

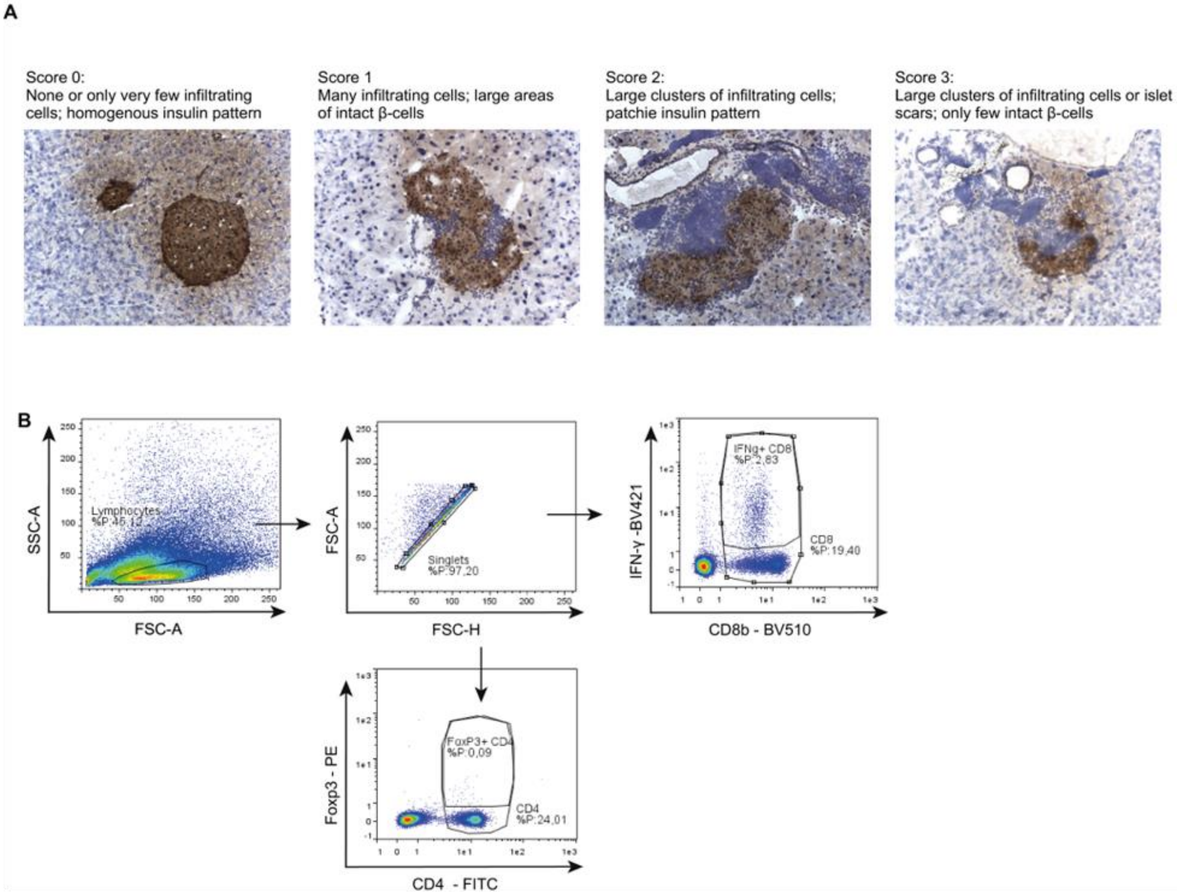

Fig. S10. Analysis of IMU-838 treatment in the virus-induced T1D RIP-LCMV-GP mouse model.

(**A**) Representative images of insulin, CD8 and hematoxylin-staining of pancreas cryosections. To evaluate the insulitis score.

(**B**) Representative FACS plots for the analysis of GP33-specific IFNγ^{+}CD8^{+} T cells and Foxp3^{+}CD4^{+} Tregs.

Table S1.

List of primers

Gene	Forward (5'-3')	Reverse (5'-3')
Histone H3	ACTGGCTACAAAAGCCG	ACTTGCCTCCTGCAAAGCAC
TCRVβ4 transgene	CATGTTTCCCTGCACATC	CCAGATCCAAAGATGAGTTGC
Rorc	GGGTGCAAGGGCTTCTTCC	CTGCTTCTTGGACATTCGGC
Dhodh	AAGATTGCCCCTGACCTCAC	GATGATGGGAATCGTGCCTTG

Table S2. Flow Cytometry Antibodies Anti-Mouse

antibody (anti-mouse)	clone	fluorophore	source
CD11b	M1/70	PB	BioLegend
CD11c	N418	BV (Brilliant Violet) 421	BioLegend
CD14	rmC5-3	V450	BD Bioscience
CD25	PC61	PerCP-Cy5.5, BV785	BioLegend
CD28	37.51	Purified	BD Bioscience
CD3	145-2C11	BV711, PE-Texas Red<br>Purified	BioLegend<br>BD Bioscience
CD4	GK1.5<br>GK 1.5<br>RM4-5<br>RM4-5	Biotin<br>FITC<br>Alexa Fluor 700,<br>PE-Cy7	BioLegend<br>Southern Biotech<br>BD Bioscience
CD44	IM7	PE<br>APC-Cy7	BioLegend<br>eBioscience/Invitrogen
CD45R/B220	RA3-6B2	PB	BioLegend
CTLA-4	UC10-4B9	PerCP-Cy5.5	Biolegend
CD62L	MEL-14	BV510, APC, PE-Cy7, BV711	BioLegend
CD8a	53-6.7	PB	BioLegend
F4/80	CI:A3-1	PB	BioLegend
Foxp3	FJK-16S	FITC, PE	Invitrogen
IFN-γ	XMG1.2	Alexa Fluor 647	BioLegend
IL-10	JES5-16E3	PE	Biolegend
IL-17A	TC11-18H10.1	BV605	Biolegend
Isotype control Rat IgG2a, κ	RTK2758	PE	Biolegend
Ki67	16A8	BV605, APC	Biolegend
PD-1	29F.1A12	PE	Biolegend
RORγt	AFKJS-9	PE	eBioscience/Invitrogen
TCRVβ4	KT4	FITC	BD Pharmingen
Strep		PB	Invitrogen

Table S3.

Flow cytometry anti-human antibodies

antibody (anti-human)	clone	fluorophore	source
CD11B	ICRF44	PB	BioLegend
CD14	HCD14	PB	BioLegend
CD19	HIB19	PB	BioLegend
CD127	A019D5	PE-Cy7	BioLegend
CD25	2A3<br>M-A251	APC<br>PE	BD Bioscience<br>BD Bioscience
CD28	28.2	Purified	BioLegend
CD3	UCHT1<br>HIT3a	Purified, BV421<br>PerCp-Cy5.5	BioLegend
CD4	RPA-T4<br>RPA-T4<br>OKT4	V500<br>BV421<br>BV785	BD Horizon<br>BD Bioscience<br>BioLegend
CD45RA	HI100<br>ALB11	FITC, AF700, BV650<br>FITC	BioLegend<br>Beckman Coulter
CD45RO	UCHL1	APC-Cy7<br>PE-Cy7	BD Pharmingen<br>BioLegend/BD Bioscience
CD8A	RPAT8<br>SK-1	PB<br>AF700	BioLegend<br>BioLegend
FOXP3	PCH101<br>236A/E7	FITC<br>PE	eBioscience/Invitrogen
KI67	16A8	BV605	Biolegend
IFNγ	4S.B3	PerCp-Cy5.5	Biolegend
IL-10	JES3-9D7	AF488	eBioscience
LAG3	11C3C65	BV711	Biolegend
TCRVβ17	IM2048	PE	Beckman Coulter
